# Unveiling the Mechanism of the *in Situ* Formation of 3D Fiber Macroassemblies with Controlled Properties

**DOI:** 10.1021/acsnano.3c00289

**Published:** 2023-03-29

**Authors:** Shiling Dong, Barbara M. Maciejewska, Maria Lißner, Daniel Thomson, David Townsend, Robert Millar, Nik Petrinic, Nicole Grobert

**Affiliations:** † Department of Materials, University of Oxford, Parks Road, Oxford OX1 3PH, U.K.; ‡ Department of Engineering, University of Oxford; Parks Road, Oxford OX1 3PJ, U.K.; § WAE Technologies Ltd, Grove, Wantage, Oxfordshire OX12 0DQ, U.K.

**Keywords:** electrospinning, sol−gel synthesis, porous fiber, phase separation, finite element analysis, oil/water separation, thermal insulation

## Abstract

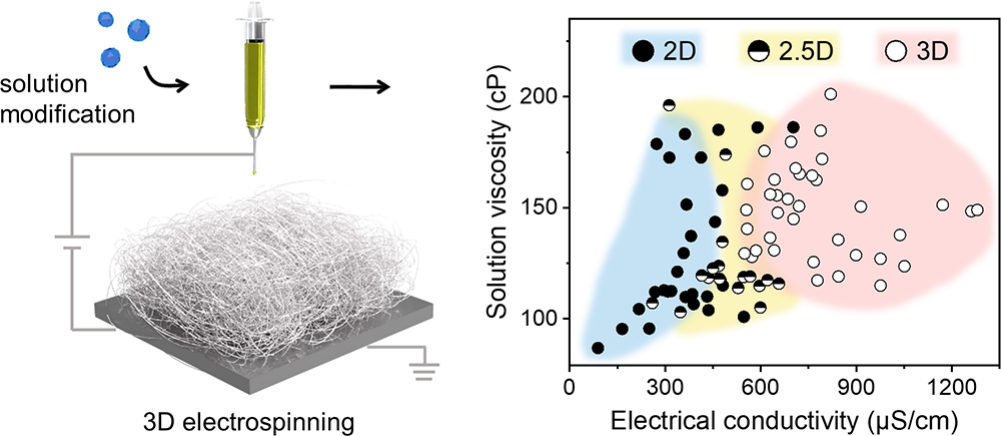

Electrospinning technique is well-known for the generation of different fibers. While it is a “simple” technique, it lies in the fact that the fibers are typically produced in the form of densely packed two-dimensional (2D) mats with limited thickness, shape, and porosity. The highly demanded three-dimensional (3D) fiber assemblies have been explored by time-consuming postprocessing and/or complex setup modifications. Here, we use a classic electrospinning setup to directly produce 3D fiber macrostructures only by modulating the spinning solution. Increasing solution conductivity modifies electrodynamic jet behavior and fiber assembling process; both are observed *in situ* using a high-speed camera. More viscous solutions render thicker fibers that own enhanced mechanical stiffness as examined by finite element analysis. We reveal the correlation between the universal solution parameters and the dimensionality of fiber assemblies, thereof, enlightening the design of more “3D spinnable” solutions that are compatible with any commercial electrospinning equipment. After a calcination step, ultralightweight ceramic fiber assemblies are generated. These inexpensive materials can clean up exceptionally large fractions of oil spillages and provide high-performance thermal insulation. This work would drive the development and scale-up production of next-generation 3D fiber materials for engineering, biomedical, and environmental applications.

## Introduction

Electrospinning is a well-established process for producing polymer-based fibers. When combined with sol–gel techniques, it allows the production of ceramic fibers in different materials, sizes, and micro- and macrostructures. In conventional electrospinning processes, the as-spun fibers are collected in the form of a densely packed fiber mat, *i.e.*, a 2D macrostructure.^1,2^ The thickness of electrospun fiber mats is usually limited to several hundreds of micrometers^3^ because the electrostatic force drives the fibers to align parallel to the collector surface while minimizing the fiber-to-fiber distance.^4,5^ These fiber mats with highly anisotropic and laminar structures usually suffer from easy delamination and poor mechanical resilience, therefore limited from application in many fields. In comparison, 3D fiber macrostructures have more diverse porosity, specific area, volume, and shape, thereof, affording more diversified functionality and better designability. They are attractive for a broad range of applications such as tissue engineering, thermal insulation, pollutant absorption, filtration, and separation.^1,6^

Early attempts to produce 3D fiber macrostructures relied on laborious postprocessing, including stacking^7^ and folding^8^ the 2D fiber mats or chopping the fiber mats into short fiber fragments and reassembling them by freeze-drying.^9,10^ However, the obtained materials are prone to disintegration due to insufficient connection and entanglement between fibers. To circumvent the time-consuming postprocessing steps, the concept of “3D electrospinning” has been proposed, *i.e.*, *in situ* creation of 3D fiber macrostructures by modifying the fiber formation, assembling, and collection processes. Significant progress has been achieved by employing 3D templates,^11,12^ replacing the metal collector with a solvent bath,^13^ and using sacrificial porogens^14,15^ to create 3D fiber macroassemblies. Yet, the obtained fiber structures were unlikely to maintain their shape and porosity after removal from the collector. Multinozzle coelectrospinning,^16^ coaxial electrospinning,^17^ near-field electrospinning jointed with 3D printing,^18^ and turbulent-flow-assisted electrospinning^19^ have resulted in 3D fiber macroassemblies with respectable structural stability and shape control, but the implementation of extra equipment not only increases the cost but also adds more unnecessarily processing parameters.

The most scalable, cost-effective, and timesaving approach toward “3D electrospinning” is keeping the conventional setup while just tuning the composition of the spinning solution, for example, adding ionic components to solutions to tailor the electrostatic interactions between fibers,^20,21^ using varied additives to tune multiple solution parameters,^22,23^ and regulating the sol–gel reaction to control the condensation between colloidal particles.^24^ However, the conclusions from these works have not been possible to extend to other material systems because of the lack of understanding of how tailoring the solution could influence the electrodynamic behavior of the solution jet and the actual assembling process of fibers. Therefore, better understanding of the mechanisms of the *in situ* formation of electrospun 3D fiber assemblies is critical to overcome the current limitation of electrospinning techniques and lead to a variety of 3D fibrous products with minimal efforts on modifying the current electrospinning setup.

Here, we modify the binary alkoxide solutions and achieve direct and rapid production of centimeter-high 3D fiber macroassemblies. The electrodynamic behavior of solution jets and the assembling process of fibers are observed *in situ* by a high-speed camera. The microstructure–mechanical property relationship of single fibers is assessed using a finite element analysis method. By modulating the sol–gel reactants to tune the solution properties, we identify a “3D region” showing the optimal ranges of solution parameters in yielding 3D fiber macrostructures. Through air calcination of the sol–gel synthesized precursor fibers, we produce ceramic fiber assemblies in both 2D mats and 3D “sponge” macroscopic forms. We demonstrate using 3D fiber assemblies as high-temperature heat insulators and absorbents for selective removal of various organic pollutants from water, showing their superiorities over the 2D counterpart.

## Results and Discussion

### From 2D to 3D Sol–Gel Electrospinning

**Figure 1:**
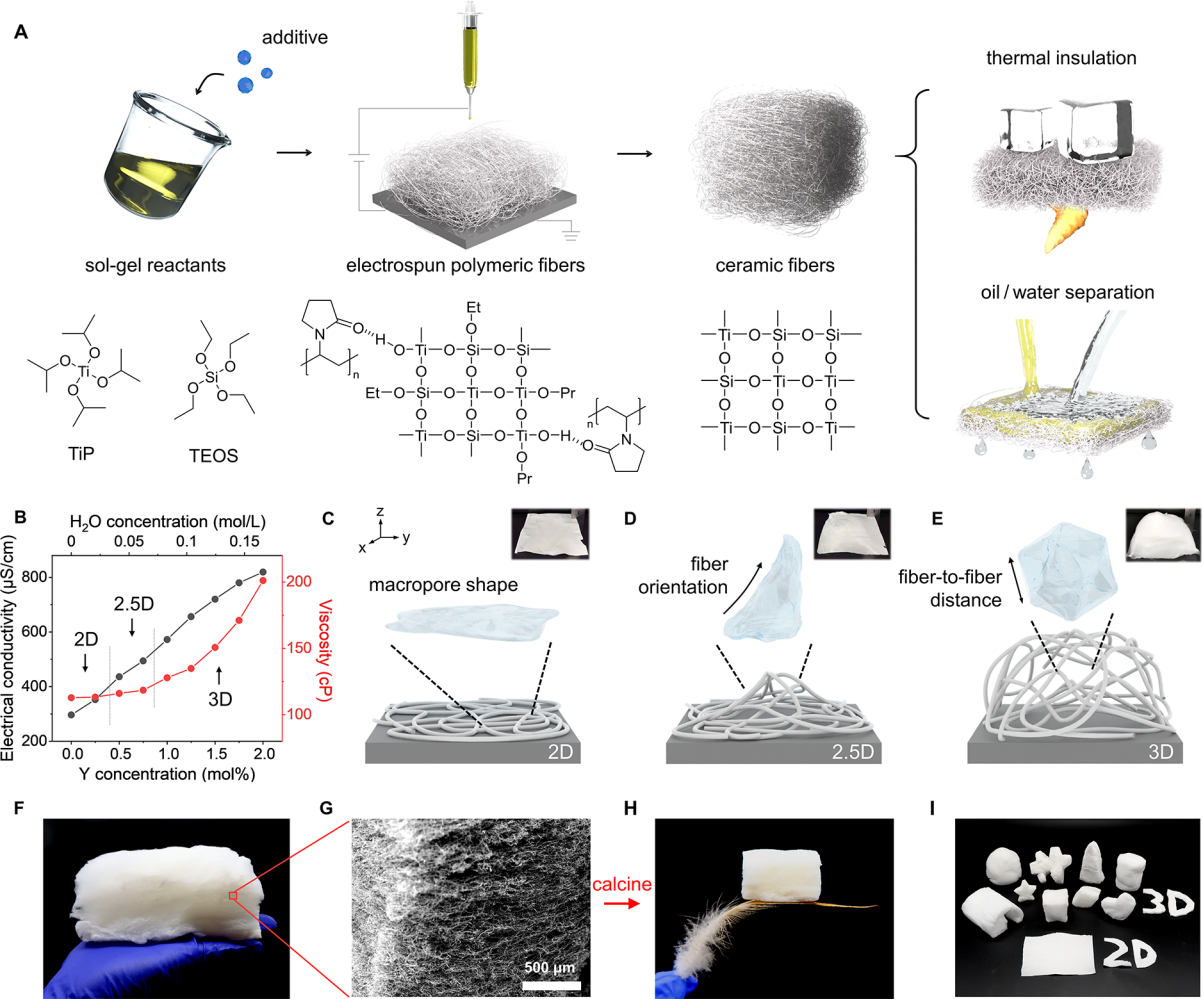
Creation of 3D fiber assembly via sol–gel electrospinning. (A) Schematic illustration of the 3D fiber assembly electrospinning process for thermal insulation and oil–water separation; the chemical composition of sol–gel reactants and solid fibers are shown. (B) The evolution of solution parameters as functions of additive concentration. (C–E) Schematics and digital photos of the 2D, 2.5D, and 3D fiber macrostructures suggest the differences in their macropore shapes, fiber orientation, and fiber-to-fiber distance. (F) Digital photo of an electrospun 3D fiber assembly from binary alkoxide solution with 2 mol % Y(NO_3_)_3_·6H_2_O. (G) SEM image shows the highly entangled and interconnected fiber network. (H) After air calcination, the ultralight ceramic fiber assembly can stand freely on a feather. (I) Photograph of 3D ceramic fiber assemblies in various shapes in contrast to the single form of 2D fiber mat.

The overall procedure of combining sol–gel synthesis with 3D electrospinning to create functional 3D fiber assemblies is depicted in Figure 1A. First, we prepared ethanol-based nonaqueous solutions containing titanium isopropoxide (TiP) and tetraethoxysilane (TEOS), common alkoxide precursors in the sol–gel synthesis of TiO_2_ and SiO_2_, respectively. Polyvinylpyrrolidone (PVP) and acetic acid (AcOH) were added as hydrolysis inhibitors. Spinning such solution resulted in 2D flat fiber mats with several hundred μm in thickness. When 0.5–2 mol % hydrated yttrium nitrate (Y(NO_3_)_3_·6H_2_O) was added to the solution, the electrospun fiber macrostructure evolved from a loosely packed 2D mat, through 2.5D architecture (coexisting 2D and 3D structure) and then to centimeter-high 3D assembly (Figure 1B–E; Supporting Information (SI) Figure S1 and Movie S1). These macroassemblies display significant differences in macropore shape, fiber orientation, and fiber-to-fiber distance along the *z*-axis, *i.e.*, perpendicular to the collector surface. The schematics highlight the change of macropore shape from anisotropic to isotropic. Because all experiments were conducted under ambient conditions using the same setup, the dimensional change originates from the altered solution properties, especially, electrical conductivity and viscosity. The electrical conductivity of binary alkoxide solutions increases linearly as a function of Y(NO_3_)_3_·6H_2_O content, explained by the increasing density of charge carriers (Y^3+^ and NO_3_^–^). Solutions with higher additive concentrations also have higher viscosity partially because the metal ions change the PVP chain configuration in the solution.^25,26^ The induced H_2_O molecules also initiate the hydrolytic sol–gel reactions of alkoxides, generating TiO_2_/SiO_2_ condensates that are more resistant to shear deformation.^27^ The formation of siloxane and metaloxane bonds and enhanced degree of condensation were confirmed by Fourier-transform infrared spectroscopy (FTIR) and thermal analysis (SI, Figures S2 and S3).

By optimizing the spinning parameters to modify the strength and uniformity of the electric field, 3D fiber assemblies in a uniform “pillow” shape were created (Figure 1F), in which the fibers are arranged into a highly interconnected 3D network without any apparent laminae (Figure 1G). The production rate of our 3D electrospinning exceeds 50 mg/min counted as the mass of fiber collected on the substrate per minute, which is much higher than the typical electrospinning works, *e.g.*, a flow rate at 1 mL/h gives less than 10 mg/min.^28,29^ Compared with previous studies,^30−34^ our 3D fiber assemblies stand out by their controllable shape, facile production, and structural integrity. After a heat treatment to remove the polymer component, Y-doped TiO_2_/SiO_2_ (TS) fibers remain long and continuous with aspect ratios >1000 (Figure S4). The fabricated ceramic fiber assembly is ultralight, flexible, elastic, and can stand on the tip of a feather (Figure 1H). Different from the single flat mat form of 2D fiber mat, such 3D fiber products own better geometry diversity and could be easily cut into varied sizes and shapes (Figure 1I).

### Electrodynamic Jet Behavior and 3D Assembly

**Figure 2:**
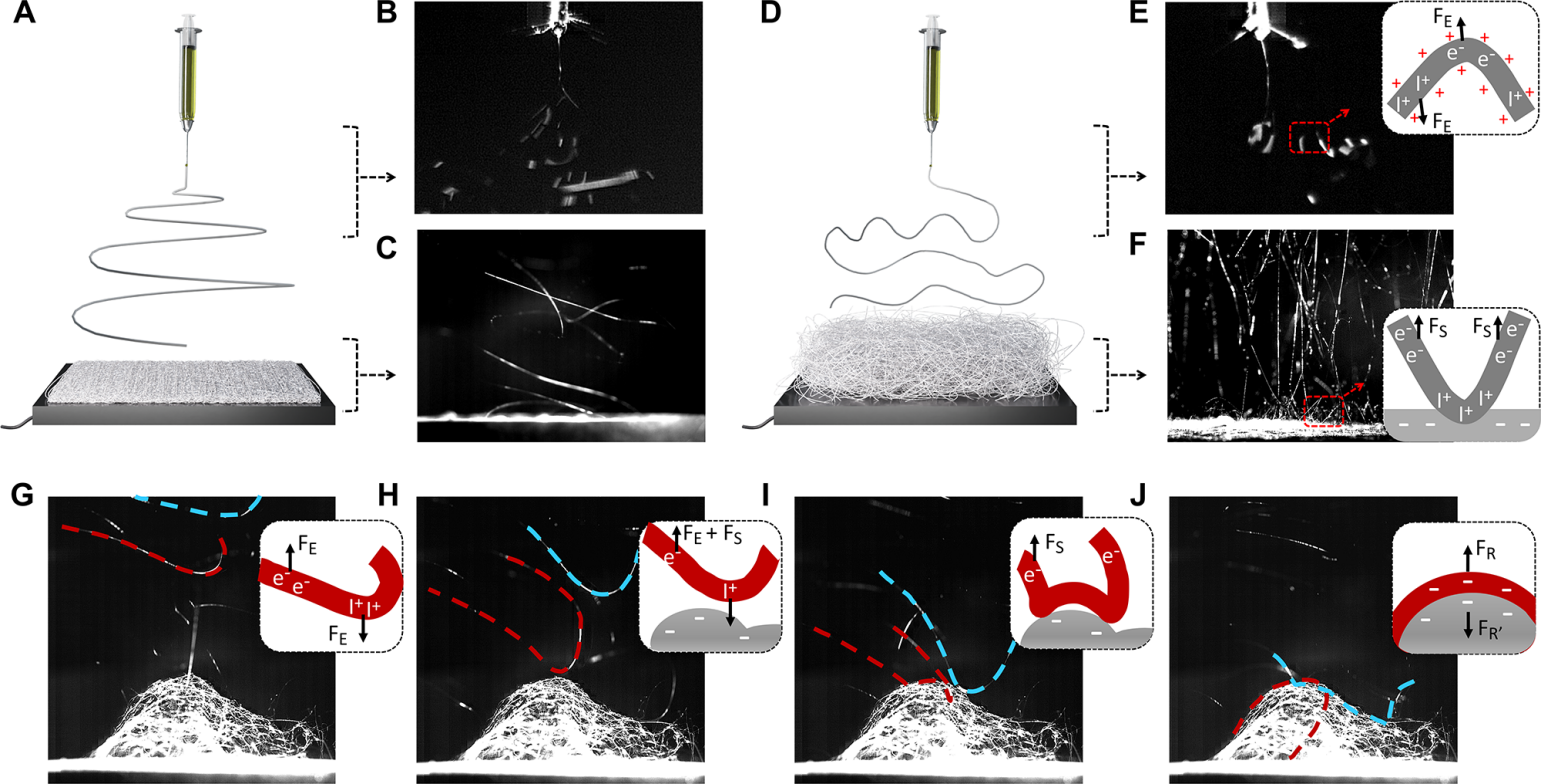
*In situ* observation of solution jet behavior and fiber assembling process. (A,D) Schematic illustrations of the 2D and 3D electrospinning processes, respectively. High-speed camera images taken near the nozzle (B,E) and at the substrate region (C,F) show the different behaviors of solution jets and solidified fibers. Inset in (E) schematically explains the formation of vertical spirals of the solution jet. The jet segment carries unevenly distributed charges. + represents the excessive charge from the high voltage. I^+^ (or e^–^) refers to positive (or negative) ions which redistribute inside the partially solidified fiber in response to the electric field. Such ions are subjected to an electrostatic force (*F*_E_) along (or opposite) the direction of electric field. Inset in (F) suggests that the top of the fiber assemblies is polarized by induced negative surface charges (white −), thus exerting repulsive force (*F*_S_) on the negative ions inside fiber to reorientate the fiber upon landing. (G–J) *In situ* observation of fiber assembling process on the substrate. Two individual fibers are traced and labeled in red and blue, respectively. Schematics highlight the forces acting on red fiber, *i.e.*, the electrostatic forces from the electric field (*F*_E_), the polarized fiber surface (*F*_S_), and the Columbic repulsions (*F*_R_), among nearby fibers once the fiber lands and undergoes polarization.

A typical 2D electrospinning process includes three stages (Figure 2A):^3^ (i) jets emerge from the solution droplet and travel along a straight path), (ii) solution jets elongate while undergoing a series of whipping and spiraling movements, and (iii) jets solidify into fibers while being collected into a dense mat. These three stages were observed in the additive-free TiP/TEOS binary solution using a high-speed camera (Figure 2B; SI, Movie S2). It is also shown in the photo that jet splitting occurs at the end of the straight jet zone which derives thinner branches. Interestingly, incorporating Y(NO_3_)_3_·6H_2_O into the spinning solution resulted in a 3D regime (Figure 2D; SI, Movie S3). In this 3D case, jet bending occurred closer to the nozzle because the solution with higher electrical conductivity has stronger Coulomb force that enhances the instability.^3,35^ The solution jets developed spirals not only vertically to the nozzle but also parallel to the electric field, which is mainly attributed to the charge redistribution.^21,36^Figure 2E schematically illustrates the proposed interaction between fiber and the electric field. The fiber segment (gray) carries excessive positive charges from the metal nozzle (red “+”), which dissipate into the environment. The ionic charge carriers, *i.e.*, NO_3_^–^, AcO^–^, and Y^3+^ (represented by e^–^ and I^+^), diffuse through the liquid phase and distribute unevenly inside the solution jet. These ions are subjected to electrostatic force (*F*_E_) either along or opposite to the direction of electric field. Such nonuniform force changes the jet conformation and leads to extra spirals at the initial stage of the bending instability zone. Note that the effect of gravity is not considered because the magnitude of gravity is negligible compared with the strong electrostatic forces present during the electrospinning process.^37,38^

In conventional 2D electrospinning, the as-spun fibers land on the collector horizontally and form a densely packed mat (Figure 2C). Distinctively, the 3D electrospun fibers were observed reaching the collector with fiber segments perpendicular to the collector surface (Figure 2F). This can be explained by electric field-induced polarization.^39^ Specifically, as the fiber approaches the substrate, it carries unevenly distributed positive and negative ionic charges. Meanwhile, the positive high-voltage induces negative charges at the top of the already collected fiber pile, making it an alternative collector for receiving the incoming fibers.^40^ The induced negative charges exert nonuniform electrostatic forces (*F*_S_) on the fiber segment, which reorient the fiber and make it land vertically on the already deposited fibers. We confirmed this by using insulators (e.g., wooden board, fabric mat) to replace the metal collector while still obtaining centimeter-high 3D assemblies (SI, Figure S5 and Movie S5). This differs from the previous studies where only flat 2D mats were obtained.^39,41^

Figure 2G–J display the step-by-step fiber assembly process with two individual fiber segments traced. The fiber reorientates close to the substrate region, followed by forming fiber entanglement in both in- and out-of-plane directions. Because the deposited fibers are subsequently negatively polarized, the Coulomb repulsive force between adjacent fibers (*F*_R_) helps maintain the large fiber-to-fiber distance.^20,42^ The attractive force between polarized fibers and metal nozzle also stabilizes the 3D architecture. This is supported by the fact that in the case of 2.5D electrospinning, fibers initially formed a 3D architecture but collapsed into 2D upon decreasing the high voltage to zero, *i.e.*, the attractive force between the polarized fibers and metal nozzle becomes weaker and finally vanished.

### Microstructure–Mechanical Property Relationship of Single Fibers

**Figure 3:**
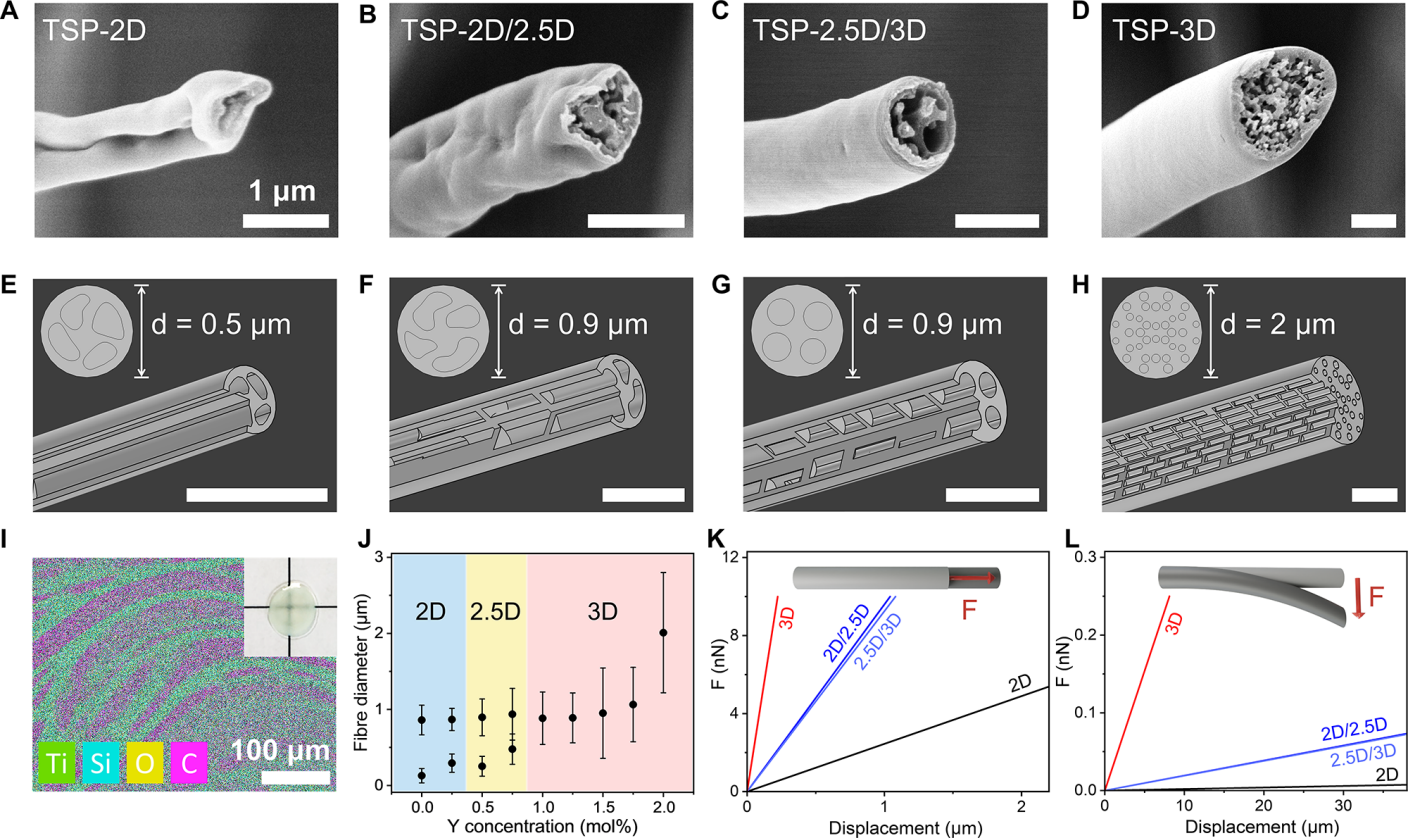
Evaluation of the stiffness of single fibers. (A–D) SEM images of the TSP fibers that are mostly populated in 2D, 2.5D, and 3D macroassemblies, respectively. For the fiber deformation simulations, four models were created with defined length (30 μm) and porosity (40%). Models of TSP-2D (E) and TSP-2D/2.5D (F) possess slit-shaped pores with irregular cross sections. Models of TSP-2.5D/3D (G) and TSP-3D (H) have cylindrical pores. (Scale bars in A–H: 1 μm.) (I) Elemental mapping on a dried droplet of binary alkoxide solution shows two phases. The inset digital photo presents the cloudiness of the droplet indicating the occurrence of phase separation. (J) The fiber size distribution versus additive concentration for each macroscopic dimension. (K,L) The simulated force–displacement curves obtained by applying a force either along or normal to the fiber axis (insets). The slopes suggest tensile and bending stiffness.

A stable 3D fiber assembly should be able to retain the large fiber-to-fiber distance and highly porous network both during electrospinning and after removal from the substrate. Such structural stability is related to the stiffness of each fiber.^43,44^ This triggered our interest in studying the microstructure–mechanical property relationship of single fibers. Figure 3A–D show the cross sections of four representative TSP fibers that exist or coexist in one or two forms of macrostructures. TSP-2D and TSP-2D/2.5D have slit-shaped pores with irregular cross sections, whereas TSP-2.5D/3D and TSP-3D have cylindrical pores with spherical cross sections. All fibers have dense shells due to the fast hydrolysis of TiP and porous cores resulting from phase separation.^45^ The two bicontinuous phases were shown by mapping the elemental distribution on a dried solution droplet (Figure 3I; SI, Figure S6). The gel-rich phase contains nonpolar TiO_2_/SiO_2_ condensates, while the solvent-rich phase has higher C content from the polar polymer PVP. Figure 3J illustrates the dependence of fiber diameters on the additive concentration. Below 1 mol % additive, TSP-2D and TSP-2.5D fibers have bimodal size distributions, including a group of microfibers and a large population of nanofibers (SI, Figure S1). TSP-3D fibers have larger average diameters because more viscous solutions have larger viscoelastic forces against jet deformation.

We used a finite element analysis method to evaluate the stiffness of single fibers, starting with building four fiber models with varied diameter (d), pore shape, and pore size matching to the observed morphologies (Figure 3E–H; SI, Figures S7, S8, details in Figure S1). A uniformly distributed force (*F*) was applied on one fiber end either along (tensile) or normal (bending) to the fiber axis (schematics in Figure 3K,L). Under the linear elastic assumption, the maximal displacement of the fiber structure is proportional to the applied force, and the slope gives stiffness. We found that TSP-2D/2.5D and TSP-2.5D/3D have similar stiffness despite varied pore design, which is consistent with the beam theory, *i.e.*, fiber diameter dominates the stiffness. TSP-3D has tensile and bending stiffness 3.9 and 16 times of the 2.5D fibers, and 18.5 and 157 times of TSP-2D, respectively, suggesting TSP-3D fiber is significantly more resilient against deformation. Since we observed that the tensile and bending stiffnesses are reversibly propositional to the first- and third-order of the fiber length, these results could be extended to longer fibers that are closer to the real case (SI, Figure S1). We further simulated the fiber deformation driven by gravity. The bending deflection of a 200 nm diameter porous fiber is 80 times that of a 2 μm diameter porous microfiber. This explains why the 2.5D macrostructures consisting of thin fibers tend to collapse due to their own weight. Overall, the analytical results suggest the dependence of the dimensionality of fiber macrostructure on the mechanical property of its building blocks, demonstrating the significance of stiffer single fibers in supporting a free-standing 3D structure.

### “3D Region” in Binary Alkoxide Solutions

**Figure 4:**
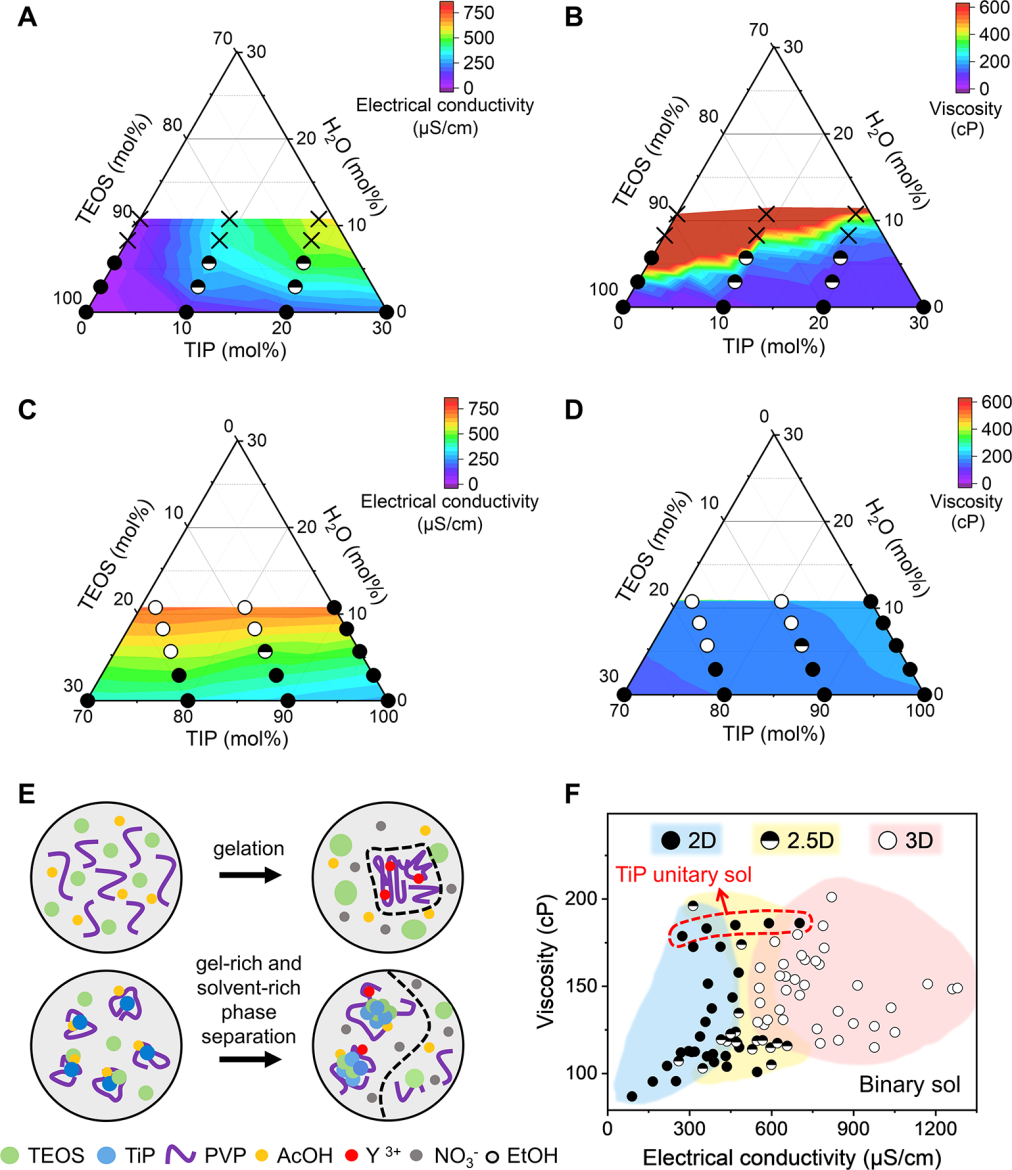
Correlation between solution and electrospun fiber macrostructure. TiP/TEOS/H_2_O ternary phase diagrams highlighting TiP-lean region (A,B) and TiP-rich region (C,D). H_2_O mol % represents 6 times of Y(NO_3_)_3_·6H_2_O mol %. The color contour displays the electrical conductivity or viscosity of each solution composition, and the symbol represents the dimension of electrospun macrostructure, ⬤ for to 2D mat, ◓ for 2.5D mixed morphology, ○ for the 3D assembly, and × for nonspinnable solutions. Schematics in (E) show the coordination of Y^3+^ to PVP chains in the TiP/TEOS 0/100 solution, which leads to local gelation of the polymer chains. The lower panel demonstrates the gel-rich and solvent-rich liquid–liquid phase separation in the TiP-rich solutions. (F) The solution viscosity and electrical conductivity are correlated to the macroscopic dimension of electrospun fibers, revealing a “3D region” for our binary alkoxide system. TiP unitary solutions are labeled by a red dotted circle due to their deviated electrospinning behavior.

We further modulated the solution by varying the TiP/TEOS ratio from 0/100 to 100/0. We created the phase diagrams by plotting the electrical conductivity and viscosity of each solution in the TiP–TEOS–H_2_O ternary phase diagrams, where H_2_O mol % corresponds to six times Y(NO_3_)_3_·6H_2_O mol % (SI, Figure S9). In the TiP-lean region (TiP ≤ 30 mol %), the electrical conductivity increases with additive and TiP concentration due to the increasing number of ion density and possible higher mobility of charge carriers (Figure 4A).^46^ The viscosity increases upon increasing additive and TEOS content until the solution turns into a gel (Figure 4B). This is because Y^3+^ coordinates to the electronegative oxygen atoms in PVP chains and creates macromolecular networks with stronger resistance against deformation (Figure 4E).^25^ In contrast, the TiP-rich solutions (TiP ≥ 70 mol %) show a linear increase in conductivity with additives (Figure 4C), while the viscosity remains almost constant or is only marginally changed (Figure 4D). This behavior is due to the reactive TiP molecules that block the electronegative sites on the PVP and prevent Y^3+^-induced gelation.^47^ Only liquid–liquid phase separation occurs in TiP-rich solutions, confirmed by the absence of cloudiness or solid sediment in the solution even after several months.

By correlating the solution parameters with the dimensions of electrospun fiber macroassemblies, we revealed a region allowing consistent creation of 3D macrostructures, named “3D region” (Figure 4F). It agrees with our earlier conclusions that high electrical conductivity is required to induce modified solution jet behavior, while moderately high solution viscosity is needed to render thicker fibers of sufficient stiffness. To reinforce the correlation, we also performed the principal component analysis (PCA) on the solution properties as shown in SI, Figure S10. The graphical distribution of data points in PCA plot is consistent with data in Figure 4F, where the solutions yielding 3D fiber macrostructures have positive PC1 scores, differentiated from the negatively scored 2D solutions, whereas the 2.5D solutions cluster at the close to 0 region as a transit stage. Moreover, we found that TiP/TEOS 100/0 solutions only generate 2D macroassembles although the solution parameters are within the “3D region”. This is because these solutions exhibit faster solidification rates and finer phase separation which inhibit both ion redistribution and the formation of porous structure (SI, Figure S11 and details in Figure S2).^48^ Similar observations were reported in emulsion electrospinning, where the oil droplets regulated the solidification process and resulted in 3D macrostructures consisting of core–shell fibers.^49^ Therefore, we anticipate that once a balance between solidification rate and charge redistribution is achieved, our proposed “3D region” is not limited to binary alkoxide solutions but suitable for various solutions even beyond the sol–gel system.

### Applications of 3D Ceramic Fiber Assemblies

**Figure 5:**
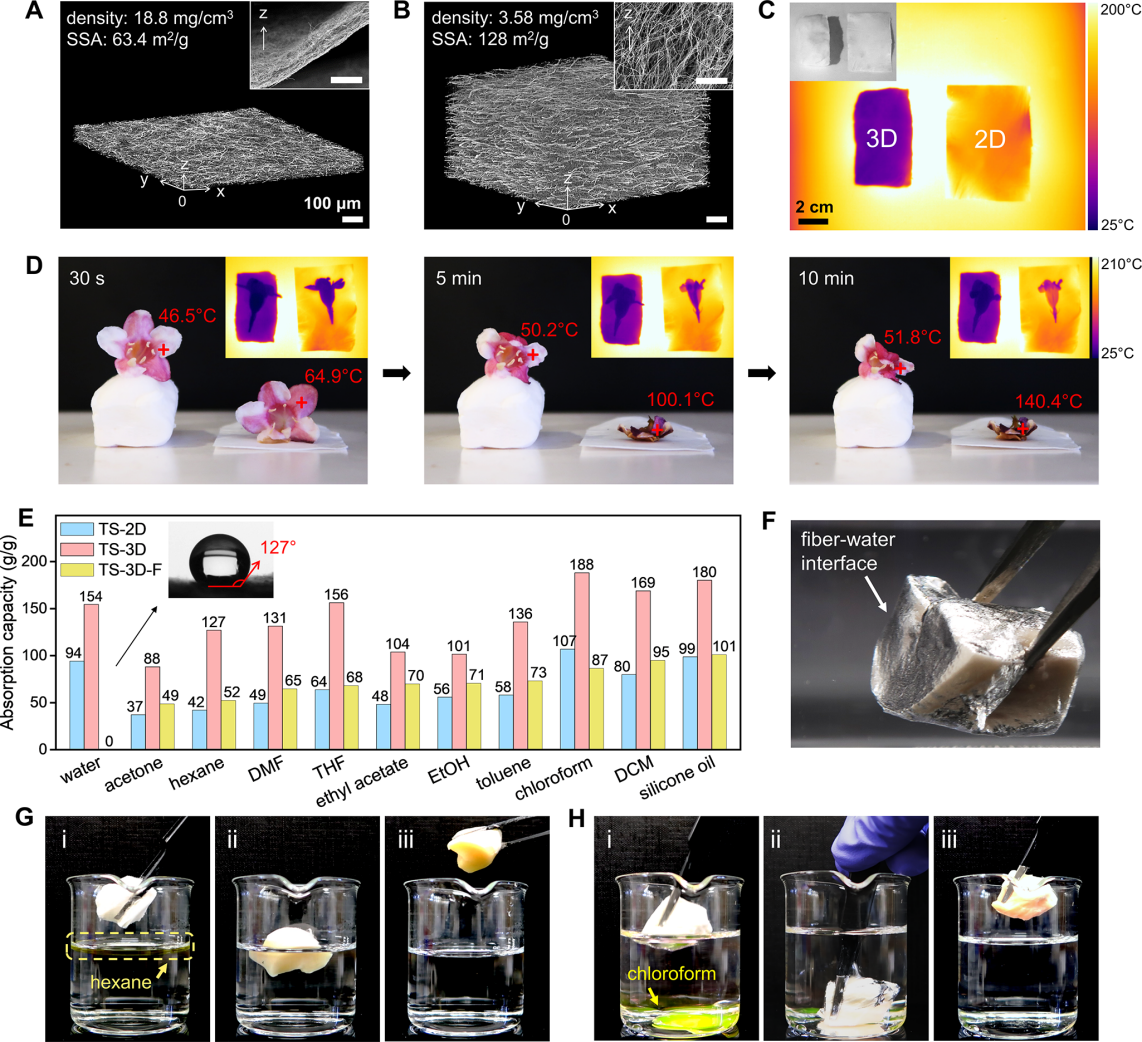
Applications of 3D ceramic fiber assembly. (A,B) 3D reconstructed XCT scans and SEM images (inset) of TS-2D and TS-3D show the different fiber arrangement, packing density, and macropore structures (scale bar: 100 μm). (C) Infrared images and photograph (inset) of TS-2D and TS-3D put side by side on a 200 °C hot plate. (D) Photographs and infrared images (inset) of two flowers placed on top of ceramic fibers. The flower on TS-3D remains fresh even after 10 min due to the much lower surface temperature. The same scale bar is used for the three infrared images (inset). (E) The absorption capacities of three types of fibers for water and several organic liquids. Functionalized TS-3D-F has a water contact angle of 127° (inset) and 0 water absorption. (F) When immersing TS-3D-F in water, the nonwetting surfaces show mirror-like reflections. (G,H) Demonstration of using TS-3D-F to selectively remove hexane and chloroform (both dyed yellow) from water.

After calcination of the TSP fibers in air, we obtained TiO_2_/SiO_2_ ceramic fibers in both forms, 2D mats (TS-2D) and 3D assembly (TS-3D). Such porous ceramic materials are very attractive for thermal insulation. X-ray computed tomography (XCT) and SEM reveal that TS-2D contains tightly packed fibers and anisotropic macropores, giving an overall density of 18.8 mg/cm^3^ and out-of-plane thermal conductivity (TC) of about 112 mW/(m·K) (Figure 5A). In comparison, TS-3D has isotropic open-cell macropores, corresponding to an ultralow density of about 3.6 mg/cm^3^ and low TC of 27.3 mW/(m·K) (Figure 5B). Both types show high thermal stability over 1000 °C (SI, Figure S12). To compare their thermal insulation performance, we placed them on a 200 °C hot plate and monitored the surface temperature using an infrared camera (Figure 5C). Upon heating at 200 °C, the 2 cm height TS-3D could protect the flower from withering for more than 10 min, while TS-2D was less effective in blocking the heat (Figure 5D). This suggests the enormous potential of TS-3D as high-temperature thermal insulator for heat protection in buildings, aircraft, aerospace, etc.

Electrospun fibers are also ideal absorbent materials. Both TS-2D and TS-3D display high absorption capacity of water and various organic solvents (Figure 5E). However, the maximum amount of liquids that TS-2D could absorb is limited by its volume. In comparison, TS-3D not only has an absorption capacity 2–3 times that of TS-2D but also can take up exceptionally large quantities of the liquid, *e.g.*, 1 g TS-3D with an overall volume of 278 cm^3^ can hold 154 mL of water. To further apply our fiber materials for the cleanup of oil spillages from the natural environment, we fluorinated the surface of TS-3D via a previously developed method^50^ to render it selectivity in absorbing, *i.e.*, being hydrophobic and oleophilic. The resultant TS-3D-F presents a large contact angle of 127° (inset in Figure 5E), and when immersing in water, mirror reflection appears on its nonwetting surface due to the entrapped air (Figure 5F). TS-3D-F has high absorption capacities of organic liquids, such as 101 g/g of silicone oil, which is approximately three times of natural sorbents and PP fiber mats.^51,52^ We show the evidence of using our as-produced TS-3D-F to selectively remove various organic solvents from water, including hexane and chloroform (Figure 5G, H), dichloromethane (DCM), silicone oil, and toluene (SI, Figure S13). Solvents with densities smaller than water, *e.g.*, hexane, can be removed by simply floating the lightweight fiber on the water surface. Solvents that are denser than water, *e.g.*, chloroform, was cleaned out by pressing TS-3D-F underwater, which rapidly absorbed the chloroform while releasing air bubbles. Full absorption was reached when the bubbling stopped. In summary, TS-3D is a powerful absorbent material with ultrahigh absorption capacity that is unachievable for 2D fiber mats. Based on that, the fluorinated TF-3D-F could separate various organic liquids from water, possessing exceptional potential for applications in environmental remediation.

## Conclusions

We showed the direct creation of 3D fiber macroassemblies and revealed the mechanism behind the 2D and 3D electrospinning regimes by a combination of high-speed camera *in situ* imaging and finite element analysis. We propose a “3D region” for binary alkoxide solutions, suggesting the optimal ranges of solution electrical conductivity and viscosity to render 3D fiber macroassemblies. Because these solution parameters are universal, our conclusions could be potentially extended to other binary and multiple component solutions beyond the sol–gel system. After calcination of the precursor fibers, we further obtained 3D ceramic fiber assemblies featured in ultralow density, high porosity, low thermal conductivity, 3D shapeability, and tunable surface wettability. Such ultralight 3D porous materials significantly outperformed 2D ceramic fiber mats in thermal insulation and oil/water separation applications. We foresee that this work could bridge the knowledge gap between 2D and 3D electrospinning and enable more functional and economical electrospun fiber materials with practical usages in insulation, environment remediation, bioengineering, etc.

## Materials and Methods

### Materials

Polyvinylpyrrolidone (PVP, MW 1 300 000), titanium(IV) isopropoxide (TiP, 97+%), and tetraethoxysilane (TEOS, 99+%) were purchased from Alfa Aesar. Yttrium(III) nitrate hexahydrate (Y(NO_3_)_3_·6H_2_O) was purchased from Fluorochem. Acetic acid (AcOH) was purchased from Honeywell. Ethanol (EtOH, ≥99.8% GC) was purchased from Sigma-Aldrich. For fluorinated coating, glutaraldehyde (GA, 50 wt % in water) and 1*H*,1*H*,2*H*,2*H*-perfluorodecyltriethoxysilane (PDTS) were purchased from Sigma-Aldrich. All reagents were used without further refinement. For measuring the absorption capacities, acetone, hexane, dimethylformamide (DMF), tetrahydrofuran (THF), ethyl acetate, dichloromethane (DCM), and silicone oil were purchased from Sigma-Aldrich. Toluene and chloroform were purchased from Fisher Scientific.

### Preparation of Ceramic Fiber Assemblies and Functionalization

The spinning solutions were generally prepared via the following steps. One g of PVP powder was dissolved in 10 mL of EtOH by continuously stirring for 1 h at 50 °C. TiP and TEOS were dropwise added into 3.7 g of PVP/EtOH solution, obtaining solution (A). Varied amounts of Y(NO_3_)_3_·6H_2_O were dissolved in 0.5 g of AcOH at room temperature, obtaining a transparent solution (B). Solution (B) was slowly added into (A), stirring at 65 °C for 3 h to obtain a clear yellowish solution, which was ready to spin after cooling down. The additive concentration was calculated as the ratio of the molar concentration of Y(NO_3_)_3_·6H_2_O to the sum up of TiP, TEOS, and Y(NO_3_)_3_·6H_2_O. Detailed solution compositions are listed in SI, Table S1.

Then 1.5 mL of each homogeneous solution was transferred to a 3 mL of plastic syringe with a metal needle (17G) attached to a high voltage supply (Genvolt High Voltage Power Supply). The solution was fed by gravitational force without further pushing. The tip-to-collector distance was maintained at 20 cm in the systematic study. The applied voltage spanned from 20 to 35 kV for optimal electrospinning continuity. Electrospinning was conducted in a glovebox with controlled environment to minimize the influence of turbulent airflow. The temperature and humidity were 22 ± 3 °C and 45 ± 15%, respectively. TiP/TEOS/PVP (TSP) precursor fibers were collected on a piece of Al foil taped on a grounded metal substrate. To convert them into ceramic fibers, heat treatments were conducted at 625 °C in air for 3 h at a heating rate of 10 °C/min, followed by natural cool down.

Fluorination was conducted on TS-3D following the procedure described in a previous report.^50^ In short, TS-3D was placed in a vacuum oven, coheated under vacuum with 1 mL of GA aqueous solution at 75 °C for 10 h, and then coheated with 250 μL of PDTS at 125 °C for 10 h.

### Characterization

The electrical conductivity of solutions was measured using a benchtop conductivity meter (Oakton CON 550) at ambient conditions (temperature 20.0 ± 0.5 °C). Each solution was measured three times and the average values were reported. The viscosity was measured by Brookfield DV-II viscometer using cone spindle CP-41. A fixed amount of 2 mL of solution was loaded into the sample cup. A ramping of rotation speed was used in 8 steps from 50 to 120 rpm, corresponding to 100–240 s^–1^ shear rate. To measure the highly viscous TiP/TEOS 0/100 solutions, the rotation speed was decreased to 10 rpm to maintain the % torque between 20 and 80%. The ramping speed viscosity measurement was conducted twice, and the averaged viscosity values were reported.

Scanning electron microscopy (SEM) images were taken using a Zeiss Merlin and a JSM-840F (JEOL Instruments) at an accelerating voltage of 4 kV. The samples were coated with 6 nm Pt before imaging. The average fiber diameters (including coating) were obtained by fitting the fiber diameter histogram based on at least 150 unbiased counts from SEM images. Energy-dispersive X-ray spectroscopy (EDX) elemental mapping was performed on dried solution droplets using a Zeiss Evo at an accelerating voltage of 5 kV. Transmission electron microscopy (TEM) images were obtained by JEOL JEM-2100F at an acceleration voltage of 200 kV. X-ray computed tomography (XCT) was conducted by a Zeiss Xradia 510 Versa X-ray microscope. The X-ray source was operated at a voltage of 50 kV and power of 4 W. The volume reconstruction was done using ImageJ in which the voxel size is 0.965 μm.

Fourier transform infrared (FTIR) attenuated total reflection (ATR) spectrum was recorded in the range of 600 to 4000 cm^–1^ on a Varian Excalibur FTS 3500 FT-IR spectrometer. Thermal gravimetry and differential thermal analysis (TG-DTA) were carried out on a PerkinElmer TG/DTA 6300 instrument in dry air from room temperature to 1000 °C with a heating rate of 10 °C/min. Brunauer–Emmett–Teller (BET) surface area was measured according to the N_2_ adsorption using a Micromeritics Gemini VII BET surface area analyzer. All the samples were degassed under vacuum for at least 10 h.

A high-speed camera (FASTCAM SA5 model) was used for the direct observation of the electrospinning process. The frame rate was 3000 or 5000 fps. The thermal conductivity was measured by a Hot Disk TPS 3500. A Kapton-insulated sensor (TPS Hot Disk 7577 with 2.0 mm in radius) was sandwiched between two TS-3D samples. The temperature distributions on the sample surfaces on the hot plate were recorded by an infrared camera (FLIR A65, FOV 45). The contact angle measurement was performed in an ambient environment using an Ossila contact angle goniometer. In the absorption tests, the fiber samples (with known weight M1) were immersed in water or organic liquids until the sample was completely wet. They were then lifted out from the liquids, held for 10 s to remove extra solvents (except for the highly volatile liquids), and then taken for weight measurement (M2). The absorption capacity (absorbency) was calculated as (M2 – M1)/M1. Each sample was tested two times for each solvent.

### Finite Element Simulation

A 3D finite element model was built using COMSOL Multiphysics 5.5. All porous fiber models have 30 μm length, 40% porosity, and randomly distributed pores with designated shapes and sizes. These fiber models have specific surface areas consistent with the BET results. Further details on the COMSOL models are given in SI, Figure SI.

The deformation of single fibers was simulated using the Structural Mechanics module. An evenly distributed load was applied to one end of the fiber model either normal or parallel to the surface to simulate uniaxial tension or bending. Young’s modulus and Poisson’s ratio of model material are 0.8 GPa and 0.32, respectively, referring to typical PVP-based soft materials.^53,54^ Three fiber models were analyzed in each case and the averaged results are presented.

## Supporting Information Available

Extended fiber simulation; solution chemistry and the formation of porous fiber structures. Figures: photos, SEM images, diameter distribution; FTIR-ATR spectra; TG/DTA curves of TSP precursor fibers; SEM images and diameter distribution of TS ceramic fibers; photos of fiber macroassemblies on varied insulating substrates; SEM and EDX mapping on a polymer solution droplet; SEM and TEM images of TS fibers; N_2_ adsorption–desorption isotherms of TSP fibers; TiP/TEOS/H_2_O ternary phase diagrams displaying the evolution of solution viscosity and electrical conductivity; principal component analysis (PCA) solution parameters; SEM images of fiber microstructure versus TiP/TEOS ratio; TC measurement and TG curve of TS fiber; extended oil/water separation experiments using TS-3D-F; simulation of the tensile and bending of a simplified fiber model at varied length; Simulation of the gravity-driven deformation of fiber models at varied diameter; schematic illustrations showing the evolution of solution jet cross sections in forming distinct fiber microstructures. Tables: Composition of alkoxide solutions analyzed for electrical conductivity and viscosity; parameters of the four types of fiber models. (PDF)
3D electrospinning on conductive Al foil substrate (MP4)
3D electrospinning on insulating wooden board (MP4)
Solution jet behavior in forming 2D fiber mat (MP4)
3D fiber assembly (MP4)
2.5D fiber structure (MP4)
