# Supplementary material for: Unveiling the Mechanism of the in Situ Formation of 3D Fiber Macroassemblies with Controlled Properties
Source: ACS Nano. 2023 Mar 29;17(7):6800–10. doi: 10.1021/acsnano.3c00289 (PMC10100559; doi:10.1021/acsnano.3c00289)
Supplement: Supplementary file 1 — nn3c00289_si_001.pdf [file nn3c00289_si_001.pdf]

Unveiling the Mechanism of the *In Situ* Formation of 3D Fiber Macro-assemblies with Controlled Properties

Shiling Dong [1], *Barbara M. Maciejewska* [1*], *Maria Lißner* [2], *Daniel Thomson* [2], *David Townsend* [2], *Robert Millar* [3], *Nik Petrinic* [2], *Nicole Grobert* [1,3*]

1 Department of Materials, University of Oxford; Parks Road, Oxford OX1 3PH, UK.

2 Department of Engineering, University of Oxford; Parks Road, Oxford OX1 3PJ, UK.

3 WAE Technologies Ltd, Grove, Wantage, Oxfordshire, OX12 0DQ, UK.

* Email: barbara.maciejewska@materials.ox.ac.uk; nicole.grobert@materials.ox.ac.uk

This PDF file includes:

Supplementary Text……………………………………………………………….………………… 2

Figure S1 to S16…………………………………………………………….………...……...… 7

Tables S1 and S2…………………………………………………………….…..……...……..… 23

Captions for Movies S1 to S5……………………..…………………………...…………… 25

Reference………………………………………………………………...……...….….…… 26

Other Supplementary Materials for this manuscript include: Movies S1 to S5

SUPPLEMENTARY TEXT

S1 Extended fiber simulation. The structural parameters of the porous fiber models are listed in **Table S2**. The porosity is calculated by

$$\frac{\text{pore volume}}{\text{solid volume} + \text{pore volume}} \tag{1}$$

A value of 40% is fixed in all models. This value is chosen based on SEM and TEM observations. The specific surface area (SSA) is calculated by

$$\frac{\text{total surface area}}{\text{solid volume} \times \text{material density}} \tag{2}$$

where the material density is 2.33 g/cm^3. The SSA of three fiber models matches BET surface areas (**Figure S8**)

Limited by the computer hardware, the four fiber models we showed in the main text have a relatively short length of 30 μm. However, in the real case, the electrospun fibers are long and continuous. To investigate the mechanical performance of longer fiber while not exponentially increasing the computation burden, we designed another series of simplified fiber models with lengths (L) from 5 μm and up to 1240 μm. These fibers are periodical structures constructed by one porous unit with 900 nm diameter and 39.8% porosity (**Figure S14 a, S14 b**). One fiber end was fixed while the other end was free. The free end was applied with a load either along the fiber

axis or vertical to it. Assuming a linear elastic material, the tensile stiffness (K_T) and bending stiffness (K_B) were derived from the force-displacement plots. **Figure S14 c** shows the stiffness against fiber length. The curve is fitted by a reciprocal function according to the theoretical definition of stiffness in a linear elastic material,

$$K_T = \frac{EA}{L} \qquad (3)$$

where E is Young's modulus and A is the cross-sectional area. Both remain constant in the periodic fiber design. The $E \times A$ value of 0.29 N μm m^{-1} was obtained.

Considering the bending of a cantilever beam (neglecting gravity), when fixing one end of the beam and applying a load at the other end, the displacement is

$$D = \frac{FL^3}{3EI} \qquad (4)$$

where I is the planar moment of inertia. E and I are invariant in the periodic fiber structure. Thus, by fitting the force-displacement curve in **Figure S14 d**, bending stiffness is given by

$$K_B = \frac{F}{D} = \frac{3EI}{L^3} \qquad (5)$$

where $3E \times I$ equals 0.06 N μm m^{-1}.

Apart from building 3D fiber models to mimic the real fiber structure, we also interchanged the pore design in TPS-2D and TPS-3D fibers, creating 2 μm-diameter fiber with slit-shaped pores

(#5) and 500 nm-diameter fiber with cylindrical pores (#6). As shown in **Figure S15 a, S15 b**, the fibers with similar diameters have comparable K_T and K_B despite the varied pore size, shape, and distribution. It suggests that for fibers with similar porosity, the stiffness is decided by fiber diameter. Furthermore, we examined the gravity-driven deformation of fibers without loading (**Figure S15 c**). A 100 μm long porous fiber with a 200 nm diameter has a bending deflection of 104 μm, which is larger than the fiber length. Such thin fibers are commonly presented in 2D and 2.5D fiber macrostructures (**Figure S1**). In comparison, the deflection of fiber with 90 nm in diameter decreases to 5.9 μm. The thicker microfiber has a smaller deflection of 1.3 μm, suggesting their better ability to maintain the shape and orientation, thus, contributing to a self-standing 3D structure.

S2 Solution chemistry and the formation of porous fiber structures. The chemical properties of solution can impact both the microstructure and macrostructure of electrospun fiber. The schematic illustration of the solidification processes of three types of solutions are depicted in **Figure S16**, which are **(a)** TiP/TEOS binary solution with additive, which undergoes 3D electrospinning, (**b**) TiP/TEOS binary solution without additive, and (**c**) TiP/TEOS 100/0 (TiP unitary solution) with additive; the latter two follow 2D regime.

At the initial stage of electrospinning, solution jets are extracted from the solution droplet and intensively stretched by electrostatic repulsion. In binary solution systems that contain two alkoxides with varied reactivities, the surface of solution jet quickly solidifies into a dense shell which inhibits further shrinkage of the fiber diameter. This enables the 'wet fiber' to carry a large quantity of liquid while flying toward the collector. Interestingly, regardless of the large variation in the fiber size from submicron to several microns in 2D, 2.5D, and 3D cases, the thicknesses of fiber shells are similarly about 100-150 nm (Figure 3). It is because the shell with a certain thickness could effectively block the permeation of moisture into the fiber interior as well as slow down the evaporation of volatile solvents. The presence of fiber shell slows down the evaporation and solidification of the solution enclosed in a fiber, enabling phase separation to develop.

Solutions that could be directly electrospun into 3D macro-assemblies possess higher viscosity. The larger viscoelastic forces against jet deformation cause thicker jets to be extracted from solution droplet and turned into thicker fibers. Comparing the 3D and 2D electrospinning processes as shown in Figure S16 a and S16 b, the thicker solution jet in the 3D case contains a larger volume of liquid in a unit length, and the solution inside further has a higher number density of mobile ions. These ions rapidly redistribute through the liquid phase, rendering jet segments positive or negative ionic charges apart from the excessive positive charge induced by the high-voltage source. While in the 2D case, the thinner solution jet has a less amount of liquid per unit length and an even smaller number density of ions due to the less electrically conductive solution. Since the thinner jet solidifies faster, the charge redistribution is limited, unable to offer the jet segments different polarity. As a result, such solution undergoes conventional 2D electrospinning process.

However, the electrospinning process of TiP unitary solutions (*i.e.,* TiP/TEOS 100/0) follows a different scenario (Figure S16 c). Even though these solutions have viscosities and electrical conductivities similar to the TiP/TEOS binary solutions known to render 3D fiber assemblies, as a result, they always yield 2D flat fiber mats which are fragile and easily cracked. [1] This is possibly attributed to the inhibited diffusion of charged species (I^+ and e^-) caused by two factors:

(i) Decreased scale of liquid-liquid phase separation. TiP reacts with the trace amount of water in the solution to generate highly reactive titanium hydroxides (Ti-OH) that rapidly attack TiP to form Ti-O-Ti. The resulted TiO_2 condensates continue to grow until all the surrounding water molecules are consumed. Due to the limited number of water molecules in the solution system, the condensates have higher number density and smaller size, which homogeneously disperse in the solution and bond to the polymer chains, making the gel-rich and solvent-rich phases finer and well-mixed. Therefore, the electrospun fibers from TiP unitary solutions are non-porous or microporous (**Figure S11**).

(ii) The solutions with higher TiP content solidify much faster because the highly reactive TiP undergoes fast hydrolysis and condensation when exposed to environmental moisture. Due to the brittleness of condensed solid TiO_2, cracks form at the fiber surface and allow quick entry of moisture into the fiber interior and escaping of the volatile solvent. Therefore, the solidification process is further speeded up, and the movement of ionic charges is greatly inhibited. With these two factors, although the solutions have high electrical conductivity and appreciable viscosity, they only result in 2D fiber mats.

Additionally, TEOS unitary solution (TiP/TEOS 0/100) also generates solid fiber (Figure S11), but in contrary to the fast solidification, this is because the slow solidification of TEOS makes the electrospun fibers still wet when reaching the collector surface. The collapsing of fiber structure vanishes all possible porous structures.

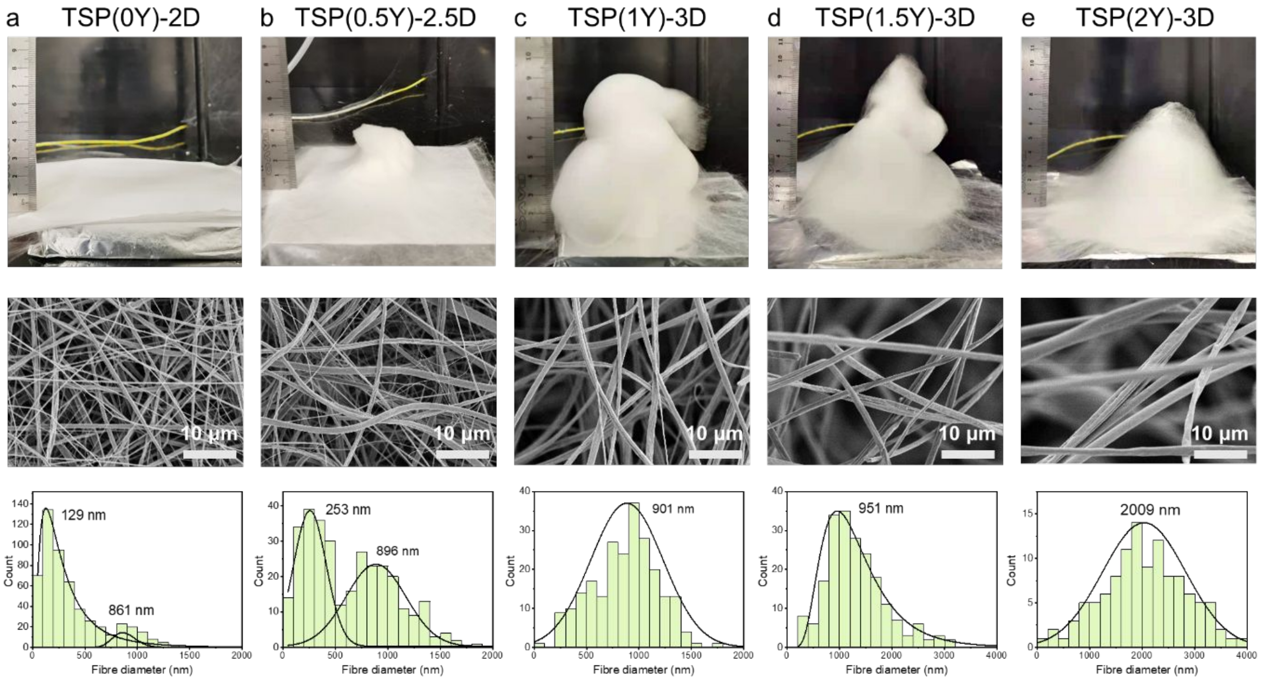

Figure S1. Digital photographs and respective SEM images of TiO_2/SiO_2/PVP (TSP) fibers electrospun from solutions with (a) 0, (b) 0.5, (c) 1, (d) 1.5, (e) 2 mol% $Y(NO_3)_3{\cdot}6H_2O$ additive concentration (denoted as 0Y to 2Y). 18-gauge nozzle was used, and the nozzle-to-substrate distance was fixed at 20 cm. The 2D, 2.5D, and 3D fiber products show significant differences on the height (or thickness) of fiber assembly and packing density of fibers. The histograms of the fiber diameters are obtained based on at least 150 counts from three different positions in each sample. The distributions were fitted by bimodal or single peak functions, and the peak positions give average fiber sizes.

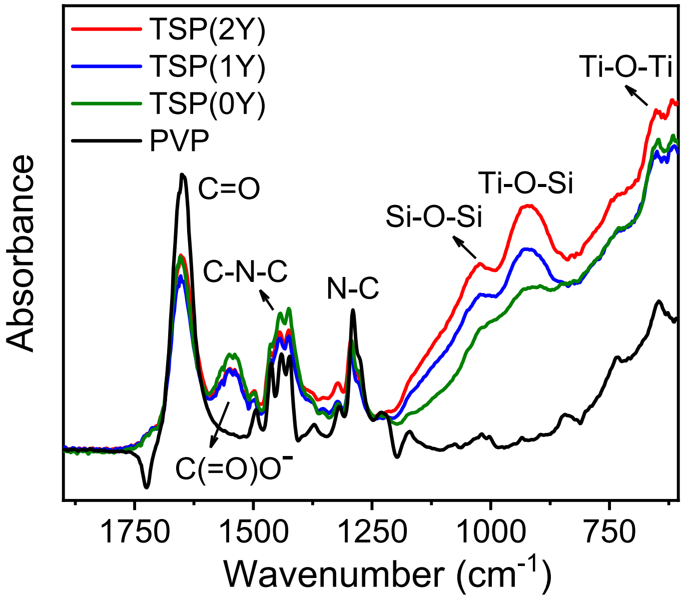

Figure S2. Fourier transform infrared (FTIR) attenuated total reflection (ATR) spectra of pure PVP fiber and TiO_2/SiO_2/PVP fibers (TSP) with varied $Y(NO_3)_3·6H_2O$ concentration (Y). The absorption peak at 1646 cm^{-1} represents the stretching mode of C=O vibration, 1463 cm^{-1}, 1439 cm^{-1} and 1424 cm^{-1} associate with the pyrrolidinyl group, and 1291 cm^{-1} and 1018 cm^{-1} indicate C-N vibrations. [2–4] TSP fibers display an absorption peak at around 1540 cm^{-1}, assigned to the protonated AcOH ($-COO^-$) from the acetate ligands in AcOH/TiP complexes and unevaporated AcOH residue. [5,6] The broad absorption band at 1100-450 cm^{-1} corresponds to siloxane and metaloxane bonds. TSP(1Y) and TSP(2Y) show high intensities of Ti-O-Ti, Ti-O-Si, Si-O-Si

peaks at 650 cm^{-1}, 790 cm^{-1}, and 960 cm^{-1} respectively, attributing to the TiO_2/SiO_2 condensates dispersed within the solution prior to electrospinning. [7,8]

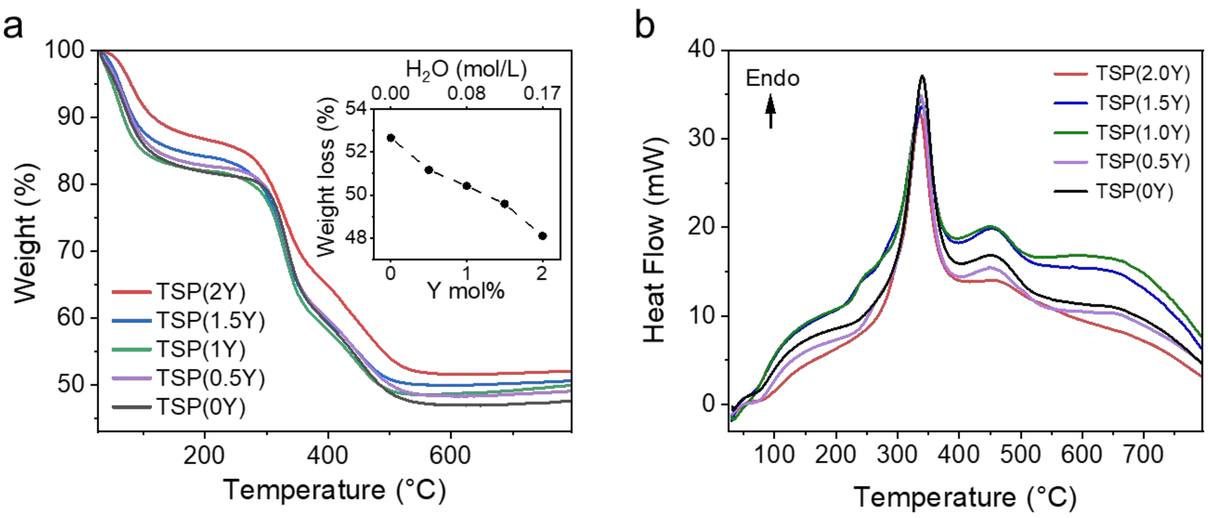

Figure S3. (a) Thermal gravimetry (TG) and (b) differential thermal analysis (DTA) curves of electrospun TSP fibers with different additive concentrations. Three weight loss stages are identified in TSP fibers: (i) 25-100 ˚C: the desorption of moisture and removal of EtOH and AcOH residues; (ii) 300-352 ˚C: the loss of PVP side groups, corresponding to the exothermic peak in DTA curve; (iii) 352-500 ˚C: the cleavage of the PVP carbon backbone. [9] Zero weight loss above 550 ˚C indicates the complete decomposition of organic components. The inset in (a) presents the weight loss against additive concentration. The total weight loss of TSP(2Y) fiber is 5% lower than TSP(0Y), confirming the higher condensation degree of alkoxides.

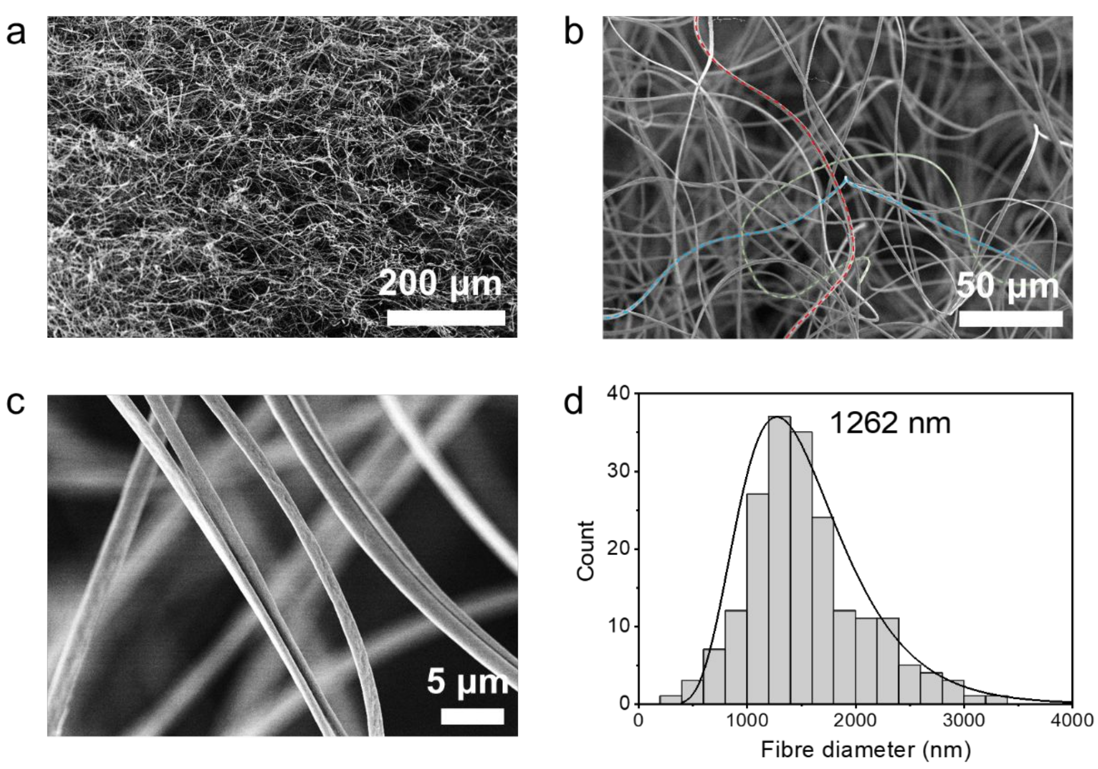

Figure S4. SEM images of TiO_2/SiO_2 (TS) fibers in different magnifications. (a) The 3D fiber assembly has highly porous architecture and isotropic open cell macropores. The colored lines in (b) highlight the long and continuous morphology of single fibers. (c) Ceramic fibers typically have homogenous diameter and slightly patterned surface. (d) Histogram of the diameter of calcined ceramic fiber with a lognormal fitting curve. The average fiber diameter decreased from about 2 μm to 1.26 μm before and after calcination.

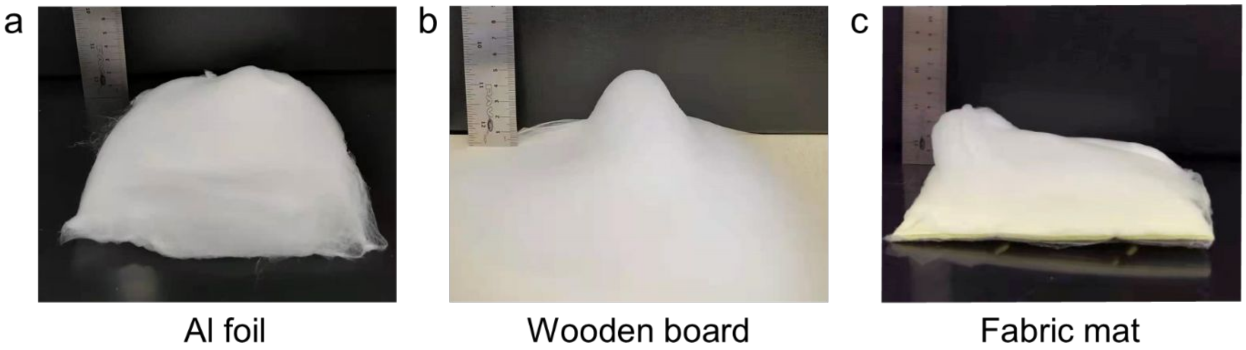

Figure S5. The fiber macrostructures obtained by electrospinning 1 mL solution on various substrates using an 18-gauge nozzle and fixing nozzle-to-tip distance at 20 cm. Although the shapes of fiber structures were changed due to the altered electric field, there was no obvious difference in the solution jet behavior and 3D fiber assembling regime. This observation validates our assumption that the surface of 3D fiber assembly is overall negatively charged due to electrostatic induction. Therefore, the as-spun fibers prefer depositing on the top layer of collected fiber, regardless of the beneath collector is conductive or insulating.

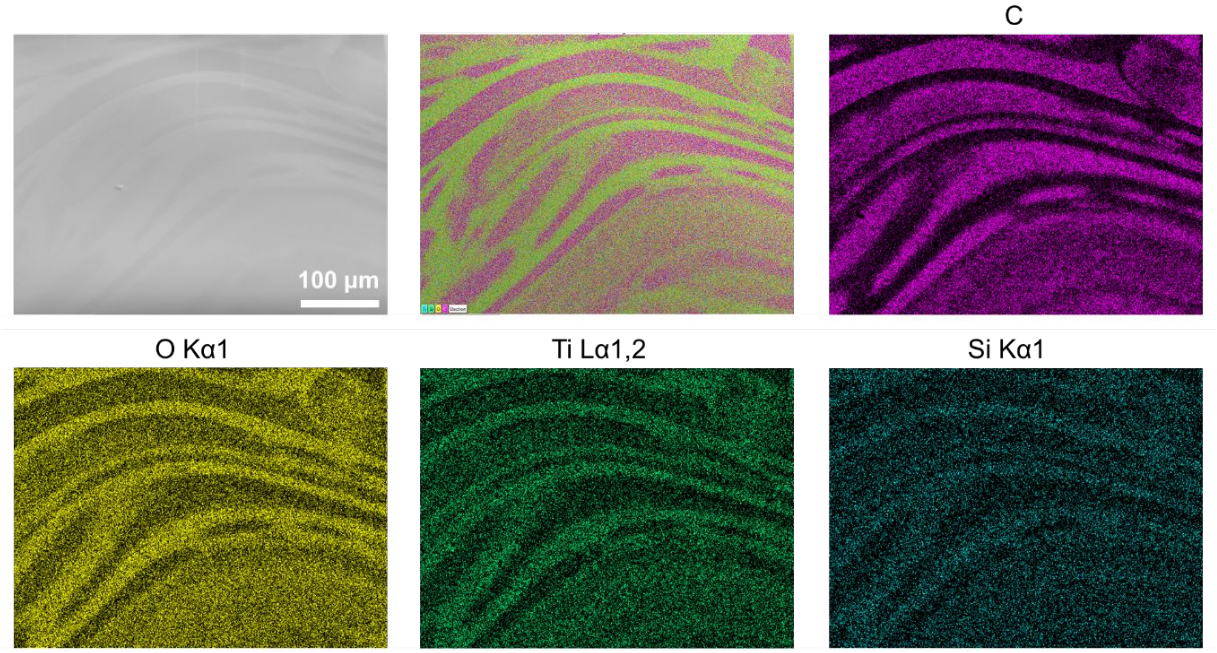

Figure S6. SEM and EDX mapping on the surface of a dried binary alkoxide solution drop without $Y(NO_3)_3{\cdot}6H_2O$ additive. Two bicontinuous regions could be identified from elemental mapping: gel-rich phase with more intensified Ti and Si signals (blue, green) due to the populated of ceramic condensates, and solvent-rich phase with higher C signal (purple) from PVP polymer.

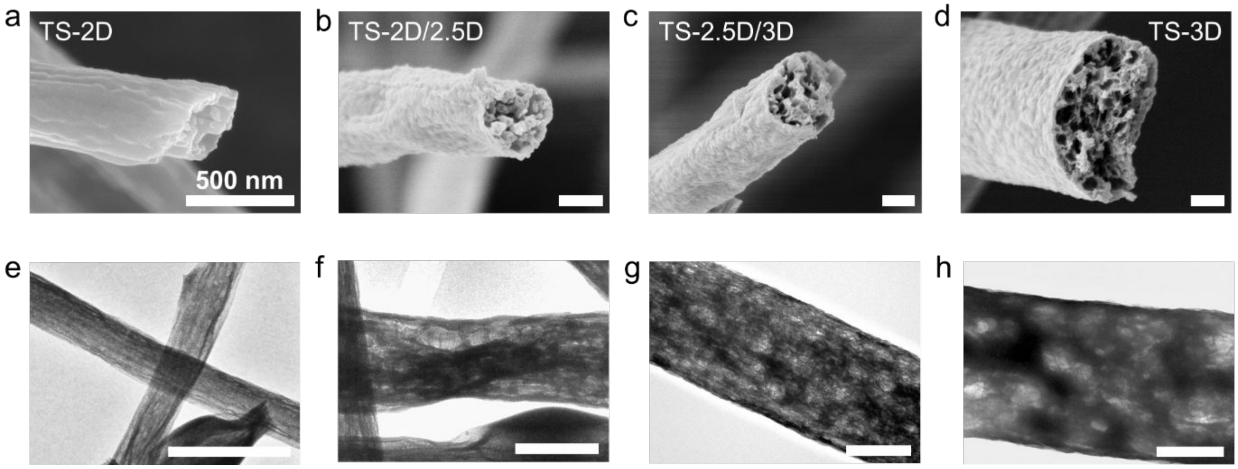

Figure S7. (a-d) SEM images of the cross-sectional area of TS ceramic fibers which are the typical building blocks in 2D mat, 2.5D architecture, and/or 3D macro-assembly. The ceramic fibers have porous core and rough shell structure. (e-h) TEM images reveal the pore distribution and pore length, which reflects the porous structure of the corresponding polymeric TSP fiber. Superficially, TSP-2D and TSP-2D/2.5D have slit-shaped pores with irregular cross-section and typical length of 2 μm and 0.9 μm respectively. The local collapse of thin shell results in a wrinkled fiber surface. TSP-2.5D/3D and TSP-3D have cylindrical pores with diameter ranging from 100-300 nm and length from 0.3-0.7 μm. Their relatively consistent diameter is due to the smaller pore size-to-fiber diameter ratio. (Scale bar in **a-h**: 500 nm)

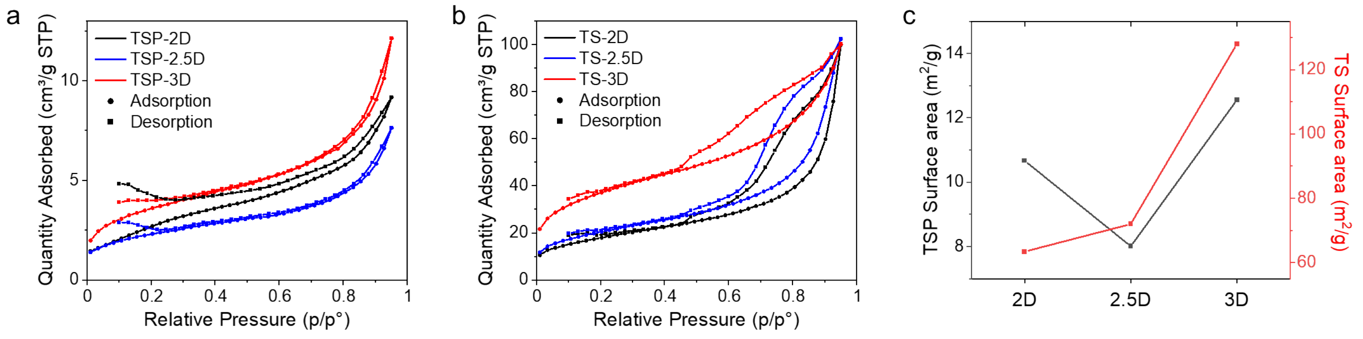

Figure S8. N_2 adsorption–desorption isotherms of as-electrospun TSP fibers (a) and calcined TS fibers (b) with different macroscopic dimensionality, *i.e.,* 2D mat, 2.5D structure, and 3D assembly. (c) Specific surface areas were obtained using a Brunauer-Emmett-Teller (BET) method.

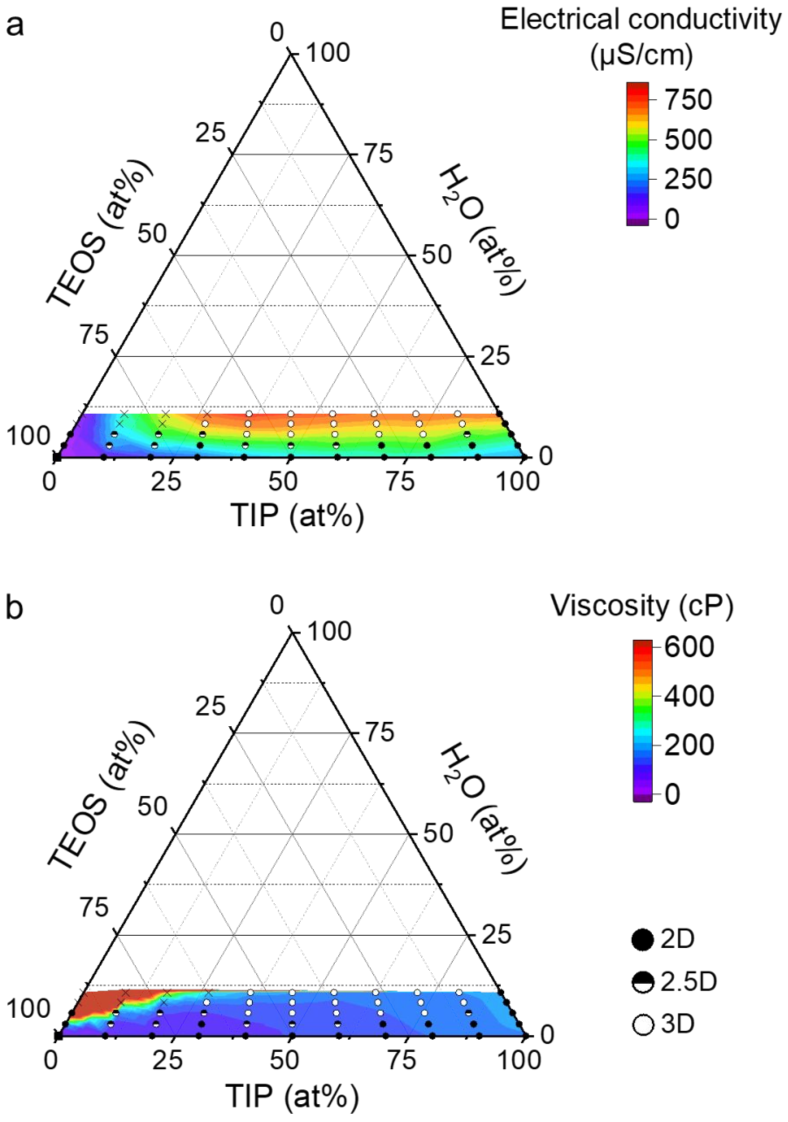

Figure S9. TiP/TEOS/H_2O ternary phase diagrams including TiP/TEOS ratio from 0/100 to 100/0. The color scale denotes the solution electrical conductivity (a) or solution viscosity (b). The circle pattern of each data point suggests the dimension of each electrospun macro-assemblies (the same as in Figure 4). × means that the solution is not spinnable.

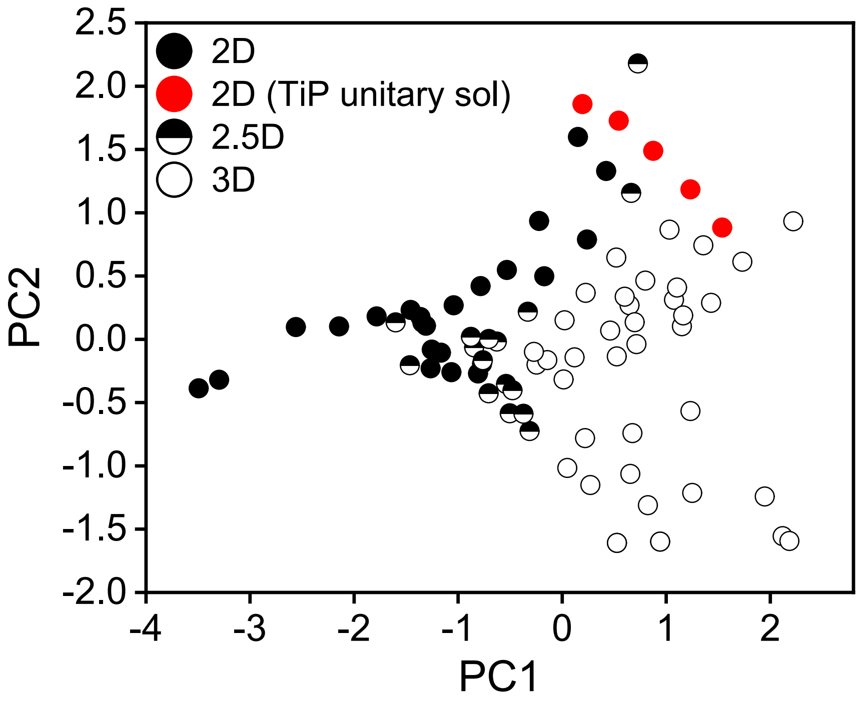

Figure S10. Principal component analysis (PCA) of 83 spinning solutions plotted versus components, PC1 (66.8%) and PC2 (33.2%). The distribution of data points distinguishes the solutions that yield fiber macrostructures with different dimensionalities. The solutions correlating to 3D assemblies are mostly located in the positive regions of PC1, *i.e.,* high electrical conductivity and high viscosity. The data points for TiP unitary solutions are labeled in red, which show 2D behavior due to the much faster solidification rate. The distinct chemical properties of these solutions make them deviate from the others.

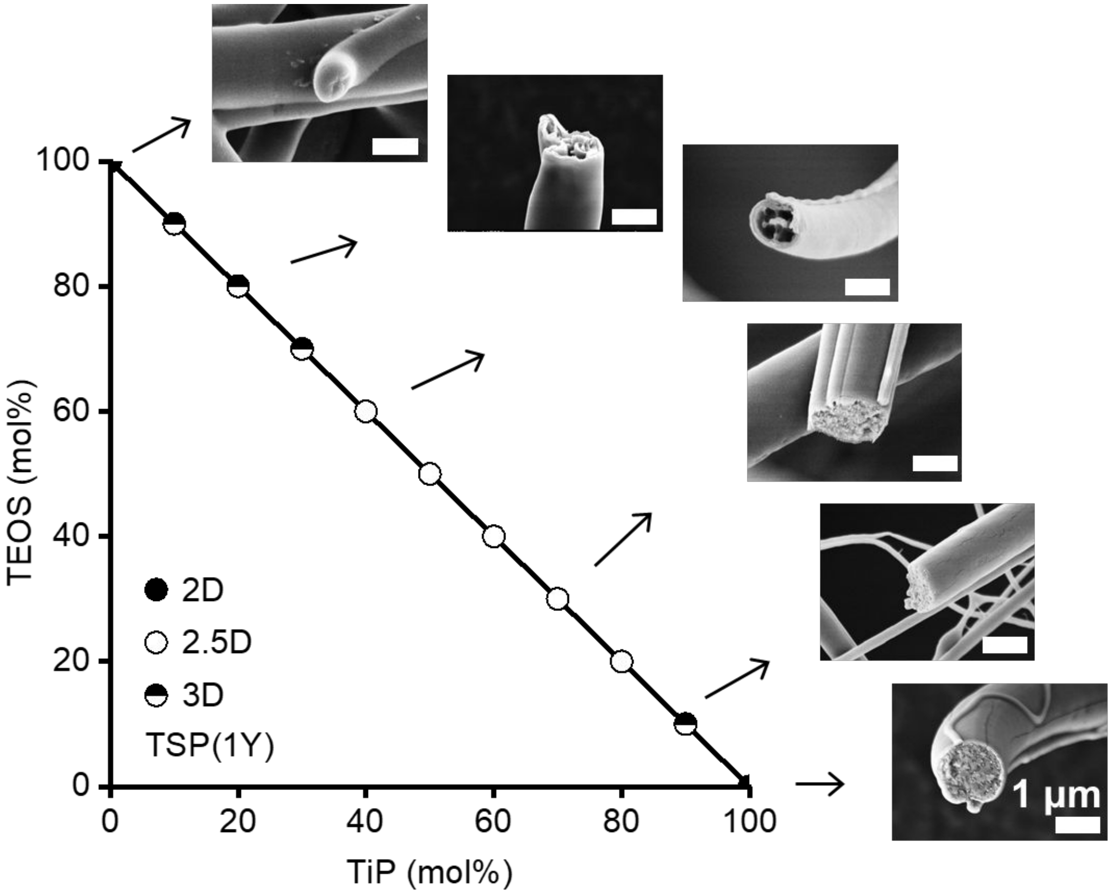

Figure S11. The evolution of fiber microstructure verses TiP/TEOS ratio at constant 1 mol% $Y(NO_3)_3{\cdot}6H_2O$ additive concentration. At TiP/TEOS of 0/100, SiO_2/PVP fibers have solid core and smooth surface, arranged in the form of flat interconnected web. TiP-lean solutions generate highly porous fibers as a result from slow solvent evaporation and the incompatibility between TEOS and PVP [10]. Increasing TiP/TEOS ratio leads to smaller pore size because the gel phase with higher TiP content solidifies faster. This leaves less time for phase separation to develop. When TiP/TEOS reaches 90/10 and 100/0, the electrospun fibers are microporous or solid, arranged into

2.5D and 2D macrostructure respectively. Cracks are visible on the fiber surfaces that cause faster gas exchange between fiber interior and environment. **(Scale bar**: 1 μm)

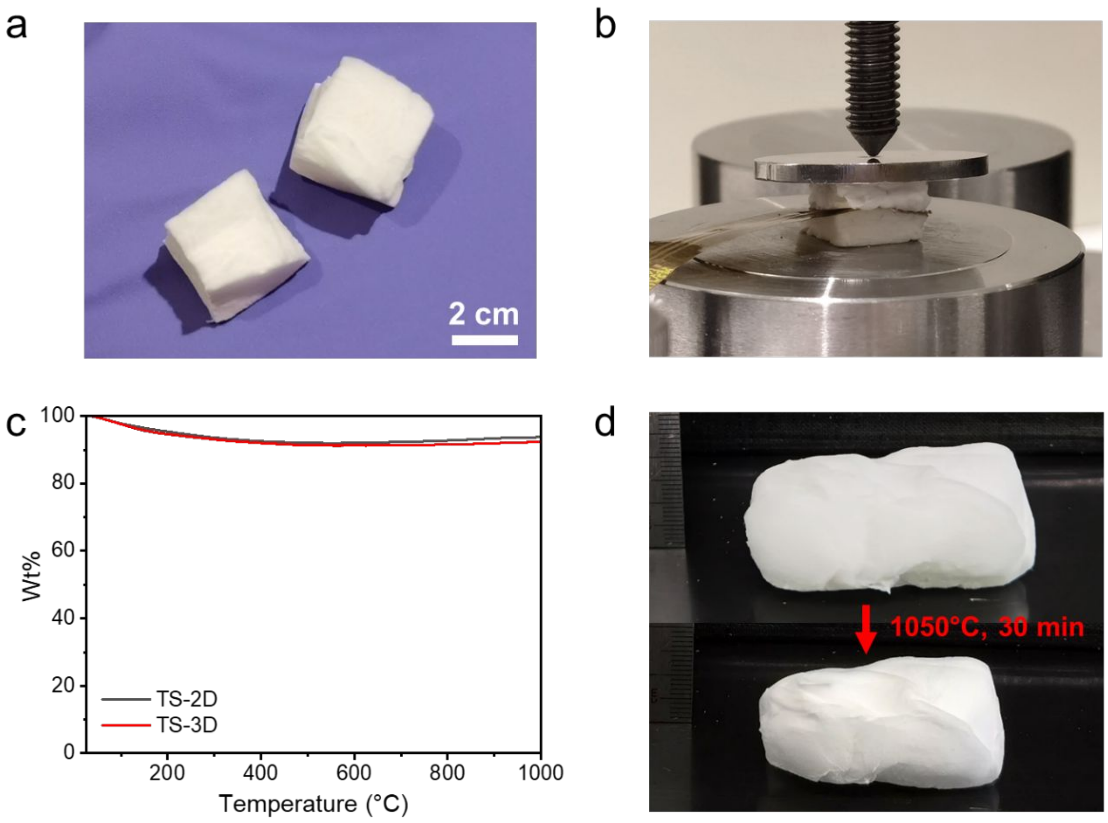

Figure S12. The thermal conductivities of ceramic fibers were measured using a transient plane source method under ambient condition. During the measurement, a hot disk sensor was sandwiched by two pieces of TS-3D in identical shape (a). The top of sample/sensor assembly was pressed by a thin metal plate to reduce thermal contact resistance (b). (c) TG curves of TS-2D and TS-3D in air atmosphere. The mass loss of about 8% from room temperature to around 500 ˚C is attributed to the evaporation of physically and chemically absorbed water. (d) The digital photos

of TS-3D before and after air calcination at 1050 ˚C for 30 min, showing the maintenance of the original shape with a slight volume shrinkage.

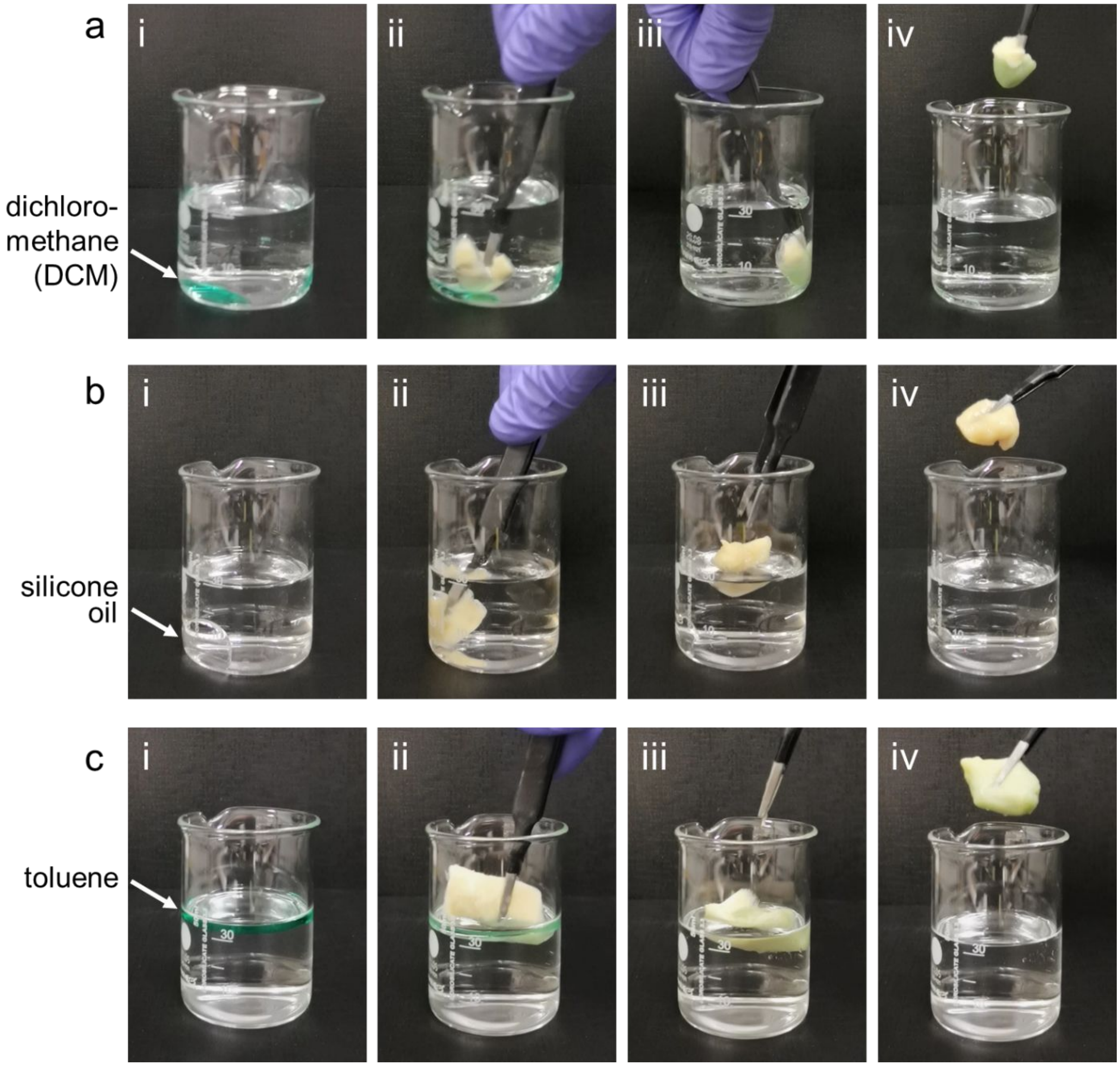

Figure S13. Demonstration of the selective removal of (a) dichloromethane, (b) silicone oil, and (c) toluene from mater using fluorinated 3D ceramic fiber assemblies (TS-3D-F).

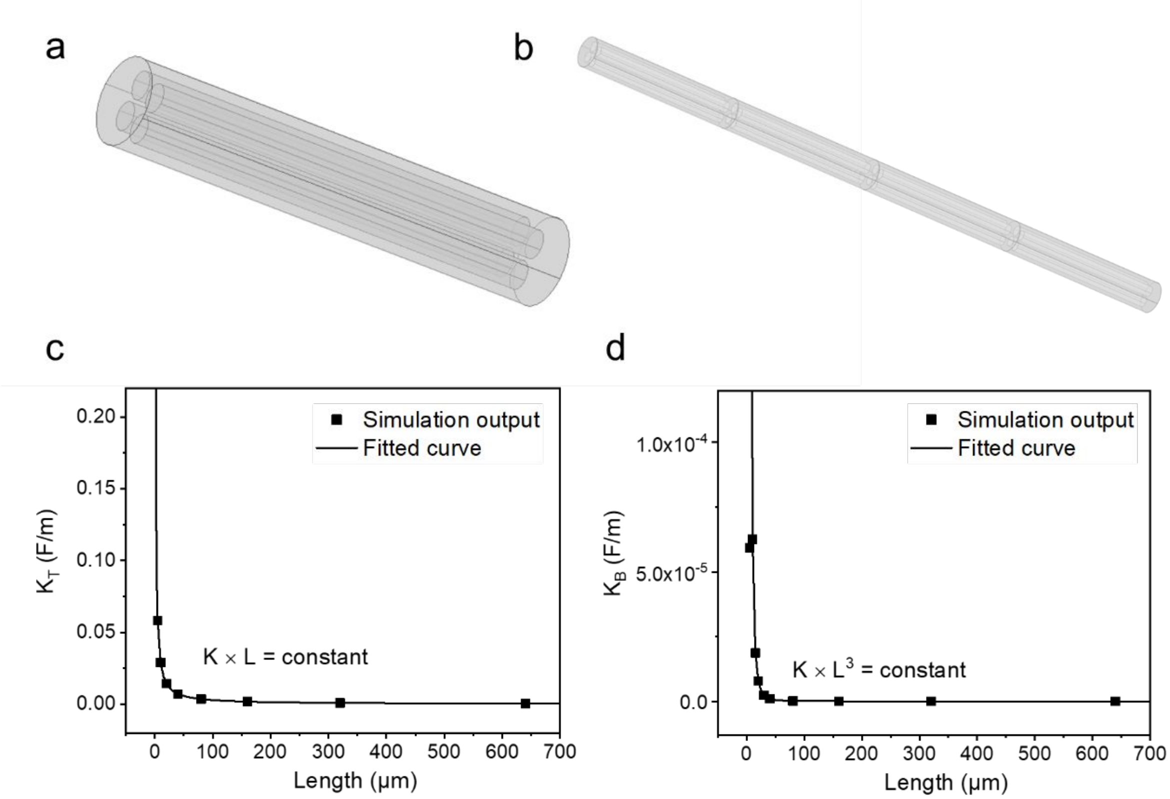

Figure S14. Simplified fiber models constructed by repeating one unit (a) to create periodic porous fiber structure, *e.g.,* four units create a length of 20 μm (b). (c, d) Tensile stiffness (K_T) and bending stiffness (K_B) of periodical fibers with varied lengths. The simulation results are fitted by functions $K_T = \mathrm{constant}/L$ and $K_B = \mathrm{constant}/L^3$ respectively.

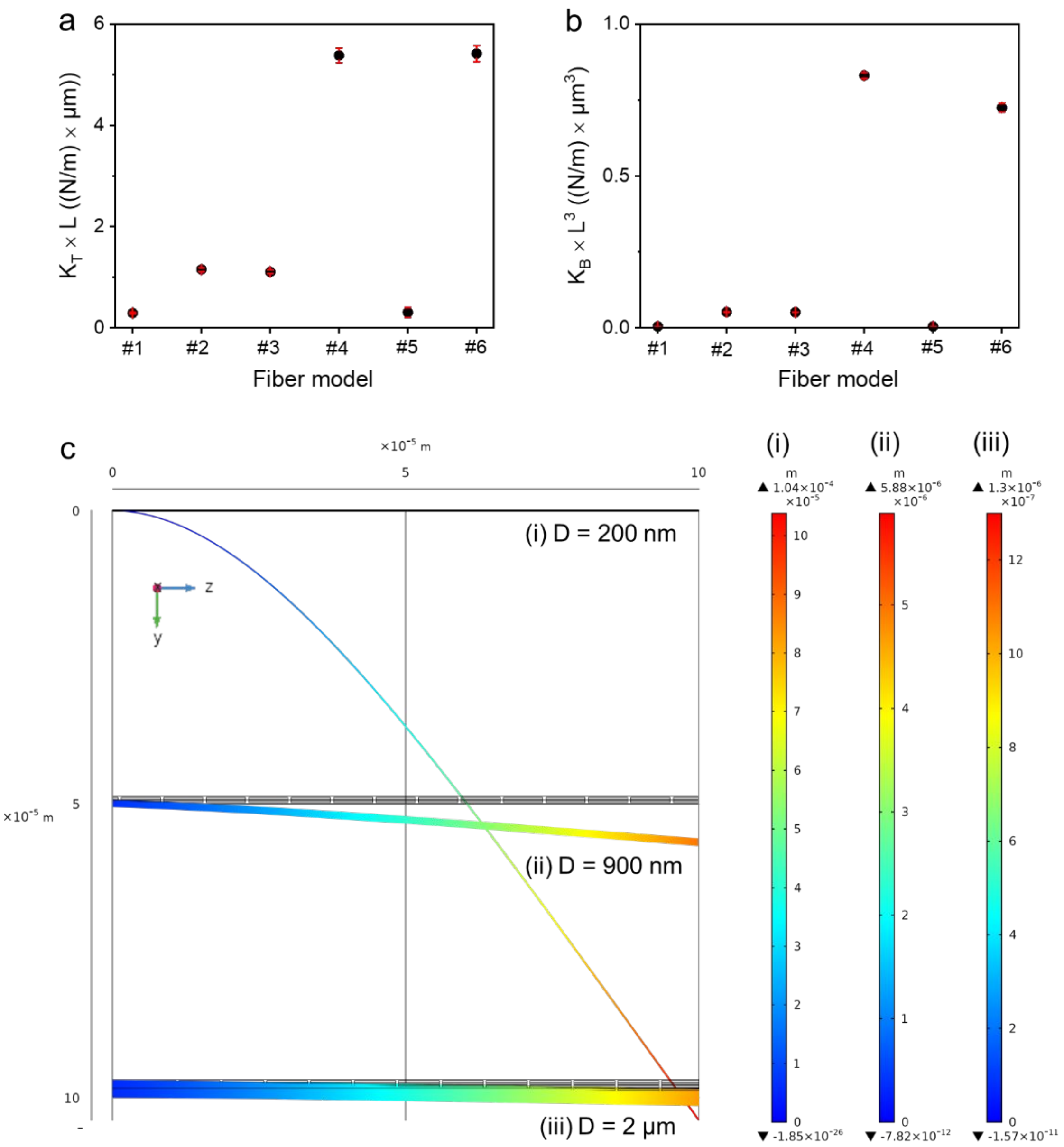

Figure S15. (a) $K_T \times L$ and (b) $K_B \times L^3$ values of each fiber model. The geometry details are given in Table S1. Error bars (in red) are calculated based on at least 5 simulated curves obtained from different fiber structures at varied length. (c) The deformation of fibers with 100 μm length due to gravity (deformation scale factor =1). The fibers have diameter (D) of (i) 200 nm, (ii) 900 nm, and

(iii) 2 μm. One end of fiber is fixed, and the gravity acts uniformly on the bottom surface. The scale bars give the structural displacement in three cases.

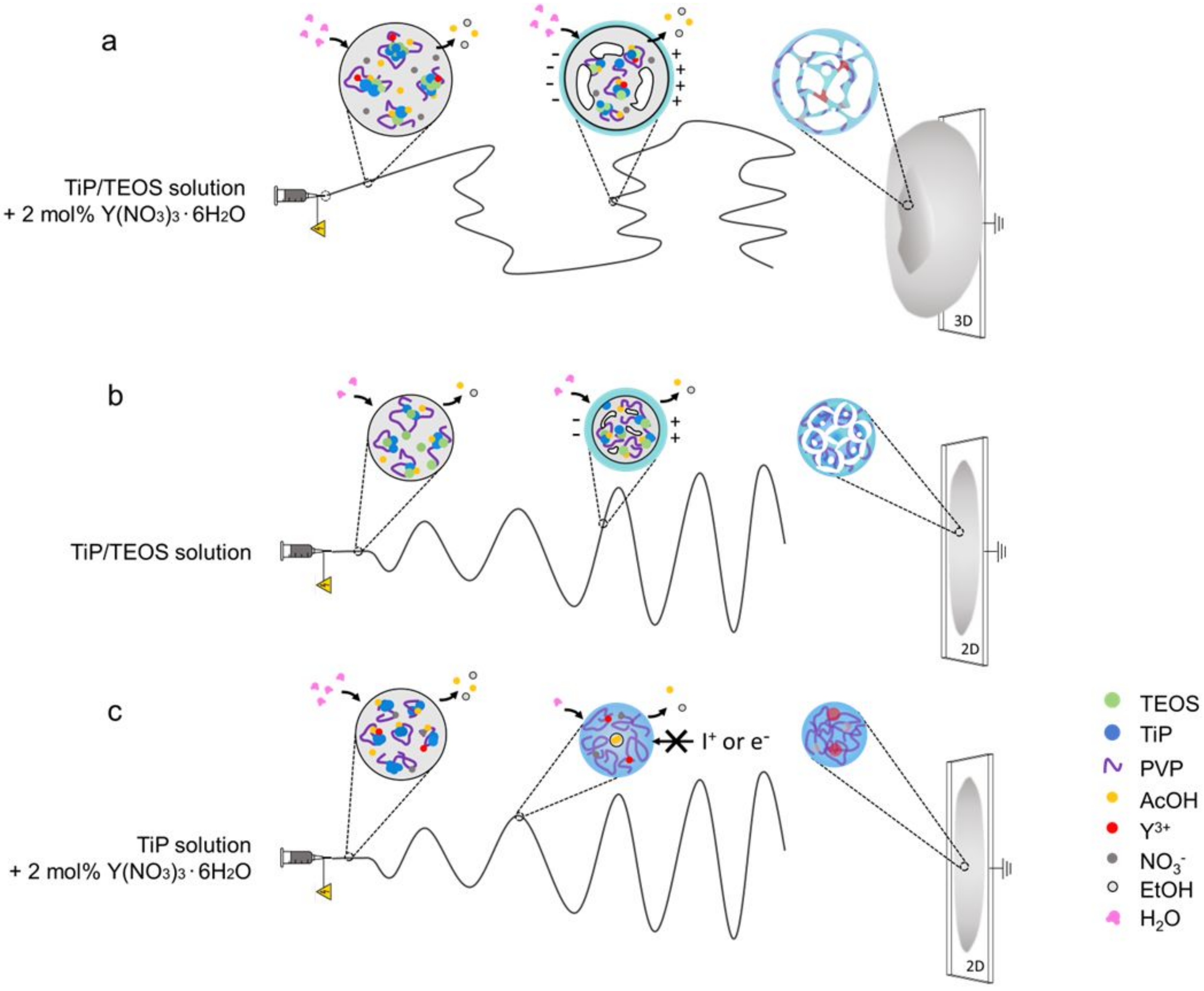

Figure S16. Schematic illustrations showing the evolution of solution jet cross-sections in forming distinct fiber microstructures. Binary solutions result in porous fiber that arranged into (a) 3D fiber assembly or (b) a 2D flat mat. (c) The unitary TiP solution results in an even thinner fiber mat due to rapid solidification rate and lack of phase separation that block the movement of charged species (I^+ and e^-).

Table S1. Composition of alkoxide solutions analyzed for electrical conductivity and viscosity.

TiP [g]	TEOS [g]	Ti/Si ratio [mol% / mol%]	$Y(NO_3)_3 \cdot 6H_2O$ [g]	Additive concentration [mol%]	Sample name and dimension
1.18	1.30	40/60	0	0	TSP(0Y)-2D
1.18	1.30	40/60	0.01	0.25	TSP(0.25Y)-2D
1.18	1.30	40/60	0.02	0.5	TSP(0.5Y)-2D
1.18	1.30	40/60	0.03	0.75	TSP(0.75Y)-2.5D
1.18	1.30	40/60	0.04	1	TSP(1Y)-3D
1.18	1.30	40/60	0.05	1.25	TSP(1.25Y)-3D
1.18	1.30	40/60	0.06	1.5	TSP(1.5Y)-3D
1.18	1.30	40/60	0.07	1.75	TSP(1.75Y)-3D
1.18	1.30	40/60	0.08	2	TSP(2Y)-3D
0	2.17	0/100	0 – 0.08	0 - 2	
0.30	1.95	10/90	0 – 0.08	0 - 2	
0.59	1.73	20/80	0 – 0.08	0 - 2	
0.89	1.52	30/70	0 – 0.08	0 - 2	
1.48	1.08	50/50	0 – 0.08	0 - 2	
1.77	0.87	60/40	0 – 0.08	0 - 2	
2.07	0.65	70/30	0 – 0.08	0 - 2	
2.36	0.43	80/20	0 – 0.08	0 - 2	
2.66	0.22	90/10	0 – 0.08	0 - 2	
2.96	0	100/0	0 – 0.08	0 - 2	

Table S2. Parameters of the four types of fiber models.

Fiber sample	Fiber diameter [μm]	Pore shape	Pore size [μm]	Pore length [μm]	Porosity [%]	SSA [m²/g]
-2D (#1)	0.5	Irregular slit	0.1-0.3	1.5-2.5	39.32	13.31
TSP-2D/2.5D (#2)	0.9	Irregular slit	0.15-0.6	0.5-1.5	39.54	7.98
TSP-2.5D/3D (#3)	0.9	Cylinder	0.15-0.3	0.3-0.5	39.21	7.94
TSP-3D (#4)	2	Cylinder	0.1-0.25	0.4-0.8	39.87	9.65
#5	0.5	Cylinder	0.1-0.25	0.4-0.8	39.98	12.70
#6	2	Irregular slit	0.1-0.3	1.5-2.5	39.75	9.49

Movie S1. The digital movie of direct electrospinning of fiber into 3D macro-assembly on a conductive plane substrate.

Movie S2. The high-speed camera observation of the solution jet behavior of (initial stage followed by overview). (Frame rate: 10 fps)

Movie S3. Solution jet behavior in forming 3D fiber assembly.

Movie S4. Solution jet behavior in forming 2.5D fiber structure.

Movie S5. 3D sol-gel electrospinning on a wooden board.

REFERENCES

(1) Fu, W.; Xu, W.; Yin, K.; Meng, X.; Wen, Y.; Peng, L.; Tang, M.; Sun, L.; Sun, Y.; Dai, Y. Flexible-in-Rigid Polycrystalline Titanium Nanofibers: A Toughening Strategy from a Macro-Scale to a Molecular-Scale. *Mater. Horizons* **2023**, *10* (1), 65–74. https://doi.org/10.1039/D2MH01255C.

(2) Someswararao, M. V; Dubey, R. S.; Subbarao, P. S. V; Singh, S. Electrospinning Process Parameters Dependent Investigation of TiO2 Nanofibers. *Results Phys.* **2018**, *11*, 223–231.

(3) Liu, H.; Zhang, B.; Shi, H.; Tang, Y.; Jiao, K.; Fu, X. Hydrothermal Synthesis of Monodisperse Ag2Se Nanoparticles in the Presence of PVP and KI and Their Application as Oligonucleotide Labels. *J. Mater. Chem.* **2008**, *18* (22), 2573–2580.

(4) Kedia, A.; Kumar, P. S. Solvent-Adaptable Poly (Vinylpyrrolidone) Binding Induced Anisotropic Shape Control of Gold Nanostructures. *J. Phys. Chem. C* **2012**, *116* (44), 23721–23728.

(5) Skotak, M.; Larsen, G. Solution Chemistry Control to Make Well Defined Submicron Continuous Fibres by Electrospinning: The (CH3CH2CH 2O)4Ti/AcOH/Poly(N-Vinylpyrrolidone) System. *J. Mater. Chem.* **2006**, *16* (29), 3031–3039. https://doi.org/10.1039/b601960a.

(6) Perrin, F. X.; Nguyen, V.; Vernet, J. L. FT-IR Spectroscopy of Acid-Modified Titanium Alkoxides: Investigations on the Nature of Carboxylate Coordination and Degree of Complexation. *J. Sol-Gel Sci. Technol.* **2003**, *28* (2), 205–215.

(7) Sadeghi, S. M.; Vaezi, M.; Kazemzadeh, A.; Jamjah, R. Morphology Enhancement of TiO2/PVP Composite Nanofibers Based on Solution Viscosity and Processing Parameters of Electrospinning Method. *J. Appl. Polym. Sci.* **2018**, *135* (23), 46337.

(8) Izutsu, H.; Nair, P. K.; Maeda, K.; Kiyozumi, Y.; Mizukami, F. Structure and Properties of TiO2-SiO2 Prepared by Sol-Gel Method in the Presence of Tartaric Acid. *Mater. Res. Bull.* **1997**, *32* (9), 1303–1311.

(9) Peniche, C.; Zaldívar, D.; Pazos, M.; Páz, S.; Bulay, A.; Román, J. S. Study of the Thermal Degradation of Poly (N-vinyl-2-pyrrolidone) by Thermogravimetry–FTIR. *J. Appl. Polym. Sci.* **1993**, *50* (3), 485–493.

(10) Wang, W.; Zhou, J.; Zhang, S.; Song, J.; Duan, H.; Zhou, M.; Gong, C.; Bao, Z.; Lu, B.; Li, X. A Novel Method to Fabricate Silica Nanotubes Based on Phase Separation Effect. *J. Mater. Chem.* **2010**, *20* (41), 9068–9072.
